# Tumour suppressors *miR-1* and *miR-133a* target the oncogenic function of purine nucleoside phosphorylase (*PNP*) in prostate cancer

**DOI:** 10.1038/bjc.2011.462

**Published:** 2011-11-08

**Authors:** S Kojima, T Chiyomaru, K Kawakami, H Yoshino, H Enokida, N Nohata, M Fuse, T Ichikawa, Y Naya, M Nakagawa, N Seki

**Affiliations:** 1 Department of Urology, Teikyo University Chiba Medical Center, 3426-3 Anesaki, Ichihara, Chiba 299-0111, Japan; 2 Department of Urology, Graduate School of Medical and Dental Sciences, Kagoshima University, 8-35-1 Sakuragaoka, Kagoshima 890-8520, Japan; 3 Department of Functional Genomics, Chiba University Graduate School of Medicine, 1-8-1 Inohana, Chuo-ku, Chiba 260-8670, Japan; 4 Department of Urology, Chiba University Graduate School of Medicine, 1-8-1 Inohana, Chuo-ku, Chiba 260-8670, Japan

**Keywords:** microRNAs, prostate cancer, *miR-1* and *miR-133a*, tumour suppressor, purine nucleoside phosphorylase (PNP)

## Abstract

**Background:**

Our recent analyses of miRNA expression signatures showed that *miR-1* and *miR-133*a were significantly reduced in several types of cancer. Interestingly, *miR-1* and *miR-133a* are located on the same chromosomal locus in the human genome. We examined the functional significance of *miR-1* and *miR-133a* in prostate cancer (PCa) cells and identified the novel molecular targets regulated by both *miR-1* and *miR-133a*.

**Methods and Results:**

The expression levels of *miR-1* and *miR-133a* were significantly downregulated in PCa compared with non-PCa tissues. Restoration of *miR-1* or *miR-133a* in PC3 and DU145 cells revealed significant inhibition of proliferation, migration, and invasion. Molecular target identification by genome-wide gene expression analysis and luciferase reporter assay showed that *purine nucleoside phosphorylase (PNP)* was directly regulated by both miRNAs. Silencing of the *PNP* gene inhibited proliferation, migration, and invasion in both PC3 and DU145 cells. Immunohistochemistry detected positive staining of PNP in PCa specimens.

**Conclusions:**

Downregulation of *miR-1* and *miR-133a* was a frequent event in PCa and both function as tumour suppressors. The *PNP* is a novel target gene of both miRNAs and potentially functions as an oncogene. Therefore, identification of novel molecular networks regulated by miRNAs may provide new insights into the underlying causes of PCa oncogenesis.

Prostate cancer (PCa) is the most frequently diagnosed cancer and second leading cause of cancer deaths among men in developed countries (Cooperberg *et al*, 2009). In early-stage PCa, >90% of patients initially respond to therapeutic use of androgen deprivation; however, many cases become refractory and progress to androgen-independent PCa (Nelson *et al*, 2008). Hormone-refractory PCa is currently difficult to treat and most clinical trials for advanced PCa have shown limited benefits, with disease progression and metastasis to bone or other sites (Bott *et al*, 2003; Timsit *et al*, 2008). Thus, new prognostic markers and effective treatment strategies are urgently needed.

MicroRNAs, small non-coding RNAs 20–22 nucleotides in length, are involved in crucial biological processes, including development, differentiation, apoptosis, and proliferation (Bartel, 2004). They pair imperfectly with target mRNAs of protein-coding genes and thereby regulate transcriptional or post-transcriptional expression (Bartel, 2009). Bioinformatic predictions indicate that miRNAs regulate >30% of the protein-coding genes (Filipowicz *et al*, 2008). It is estimated that ∼1000 miRNAs exist in the vertebrate genome. At present, 1424 human miRNAs have been registered at miRBase release 17.0 (http://microrna.sanger.ac.uk/). A growing body of evidence indicates that miRNAs contribute to the initiation and development of many types of human cancer (Zhang *et al*, 2007). Overexpressed miRNAs could act as oncogenes by repressing tumour suppressor genes. Analogously, miRNAs which normally suppress oncogenes could permit their function when underexpressed (Davis and Hata, 2010).

Many genome-wide profiling studies have been carried out to identify differentially expressed miRNAs (Lu *et al*, 2005; Calin and Croce, 2006). In comparative miRNA expression studies, several miRNAs (e.g., the *miR-34* family, the *miR-200* family, *miR-101*, *miR-15a/16*, *miR-205*, *miR-146,* and *miR-145*) were often found to be downregulated. Those observations suggested that these miRNAs could be important in PCa initiation and development (Catto *et al*, 2011). Functional analyses of these miRNAs have been conducted at many research facilities. Furthermore, miRNA profile in PCa provides evidence that miRNAs can be used as diagnostic and prognostic markers (Schaefer *et al*, 2010). More recently, we and other researchers demonstrated that restoration of *miR-145* inhibited cell proliferation, migration, and invasion in PCa cells (Zaman *et al*, 2010; Fuse *et al*, 2011). We performed genome-wide gene expression analysis to identify candidate genes targeted by *miR-145*, and several oncogenic genes were identified, such as FSCN1 and SWAP70 (Chiyomaru *et al*, 2011; Fuse *et al*, 2011). The identification of tumour suppressive miRNAs and their target genes could provide new insights into potential mechanisms of PCa oncogenesis.

Recently, our miRNA expression signature analyses showed that *miR-1* and *miR-133a* were significantly reduced in several types of cancer (Kano *et al*, 2010; Yoshino *et al*, 2011; Nohata *et al*, 2011a). Furthermore, overexpression of both miRNAs inhibited cancer cell proliferation and induced cancer cell apoptosis (Nasser *et al*, 2008; Chiyomaru *et al*, 2010b; Kawakami *et al*, 2011; Yoshino *et al*, 2011; Nohata *et al*, 2011b). Interestingly, *miR-1-1/miR-133a-2* and *miR-1-2/miR-133a-1* are clustered on different chromosomal regions in the human genome, 20q13.33 and 18q11.2, respectively. It is well known that several miRNAs form a cluster in the human genome such as *miR-17-92*, *miR-106a-368*, *miR-221-222*, *miR-106b-25* and the *miR-200* family. In PCa oncogenesis, the *miR-15a/16-1* cluster acts as a tumour suppressor by targeting multiple oncogenes, including *BCL2*, *CCND1*, and *WINT3A* (Bonci *et al*, 2008).

We hypothesised that *miR-1* and *miR-133a* might be previously unidentified tumour suppressive miRNAs in PCa. The next challenge was to identify *miR-1*- and *miR-133a-*regulated cancer pathways. For target genes searches of *miR-1* and *miR-133a* in PCa cells, we performed genome-wide gene expression analyses. We focused on the *purine nucleoside phosphorylase (PNP)* gene as a candidate target of *miR-1* and *miR-133a* in PCa cells. Insights into the association between tumour suppressive miRNAs and their target oncogene networks could enhance our understanding of the molecular mechanism of PCa oncogenesis.

## Materials and methods

### Clinical prostate specimens

The clinical specimens were obtained from patients in Teikyo University Chiba Medical Center Hospital from 2008 to 2010. All the patients had elevated levels of prostate-specific antigen and had undergone transrectal prostate needle biopsy. Prostatic cancerous tissues (PCa, *n*=15) and non-cancerous tissues (non-PCa, *n*=17) were used in this study. The patients’ characteristics are shown in Table 1 and Supplementary Table 1. Written consent of tissue donation for research purposes was obtained from patients before tissue collection. The protocol was approved by the Institutional Review Board of Teikyo University. To justified tissue composition, a pair of needle biopsy specimen was collected from same region in the patients in this study, and one was subjected to pathological justification; no cancerous tissue was found in specimens of Non-PCa.

### Cell culture

We used two human PCa cell lines, PC3 and DU145, which were obtained from the American Type Culture Collection (Manassas, VA, USA) and were maintained in RPMI-1640 medium supplemented with 10% fetal bovine serum in a humidified atmosphere of 5% CO_2_ and 95% air at 37°C.

### RNA extraction

Total RNA including miRNA was extracted using the mirVana miRNA isolation kit (Ambion, Austin, TX, USA) using the manufacturer's protocol. The integrity of the RNA was checked with the RNA 6000 Nano Assay Kit and a 2100 Bioanalyzer (Agilent Technologies, Santa Clara, CA, USA). Normal prostate RNAs were purchased as follows: prostate 1 (Clontech, Mountain View, CA, USA; Human Prostate Total RNA, no. 636550), prostate 2 (FirstChoice Human Prostate Total RNA, #AM7988, Applied Biosystems, Foster City, CA, USA), and prostate 3 (Biochain Total RNA-Human adult Normal Tissue, Hayward, CA, USA; no. R1234201-P).

### Quantitative real-time RT–PCR

First-strand cDNA was prepared from total RNA (1 *μ*g) using a High Capacity cDNA Reverse Transcription Kit (Applied Biosystems). In the real-time PCR step, complementary DNA was amplified and the gene-specific PCR products were assayed using the 7900-HT Real-Time PCR System according to the manufacturer’s protocol. Thermal cycling conditions were as follows: 95°C for 10 min, 40 cycles of 95°C for 15 s and 63°C for 1 min. TaqMan probes and primers for *PNP* (assay ID: Hs00165367_m1) and human *GAPDH* (assay ID: Hs0329097_g1) were obtained from Applied Biosystems (Assay-On-Demand Gene Expression Products). For miRNA RT–PCR, the cDNA strand was synthesised using TaqMan quantitative real-time PCR (TaqMan MicroRNA Assay; Applied Biosystems). The expression levels of *miR-1* (assay ID: 002222) and *miR-133a* (assay ID: 002246) were normalised to *RNU6B* (Assay ID 001973). All reactions were performed in triplicate, and negative controls lacking cDNA were included. The data were analysed with the delta–delta Ct method to calculate the fold change. About the internal controls, we examined the unevenness between the clinical samples and adopted to use *GAPDH* and *RNU6B* in this study.

### Mature miRNA and siRNA transfection

Pre-miR and negative-control miRNA (Applied Biosystems) were used in the gain-of-function experiments, whereas *PNP* siRNA (Cat # HSS107263 and HSS181558; Invitrogen, Carlsbad, CA, USA) and negative-control siRNA (D-001810-10; Thermo Fisher Scientific, Waltham, MA, USA) were used in the loss-of-function experiments. As previously described (Ichimi *et al*, 2009), PC3 and DU145 cells were transiently transfected with either precursors of *miR-1* and *miR-133a* or negative control using Lipofectamine RNAiMAX transfection reagent (Invitrogen), according to the manufacturer's recommendations. Mock transfections, which only had the transfection reagent, were also used as controls. The transfection efficiency of miRNA into cancer cells was evaluated by downregulation of mRNA levels of *PTK9* as described previously (Ichimi *et al*, 2009). Cells were seeded in 6-well plates for the mRNA and protein extraction and the wound-healing assays (25 × 10^4^ per well), in 24-well plates for luciferase reporter assays (10 × 10^4^ per well), and in 96-well plates for XTT assays (3000 per well).

### Cell proliferation, migration, and invasion assays

Cell proliferation was determined by using an XTT assay (Roche Applied Sciences, Tokyo, Japan) performed according to the manufacturer's instructions. Cell migration activity was evaluated with a wound-healing assay. Cells were plated in 6-well dishes, and the cell monolayers were scraped using a P-20 micropipette tip. The initial gap length (0 h) and the residual gap length 24 h after wounding were calculated from photomicrographs. A cell invasion assay was carried out using modified Boyden Chambers consisting of transwell-precoated Matrigel membrane filter inserts with 8 *μ*m micron pores in 24-well tissue culture plates (BD Biosciences, Bedford, MA, USA). Minimum essential medium containing 10% fetal bovine serum in the lower chamber served as the chemoattractant, as described previously (Chiyomaru *et al*, 2010b). All experiments were performed in triplicate.

### Western blot analysis

After 3 days of transfection, protein lysates (30 *μ*g) were separated on NuPAGE on 4–12% bis-tris gels (Invitrogen) and transferred onto a polyvinylidene fluoride membrane. Immunoblotting was done with diluted (1 : 500) polyclonal PNP antibody (HPA001625; Sigma-Aldrich, St Louis, MO, USA) and GAPDH antibody (MAB374; Chemicon, Temecula, CA, USA). The membrane was washed and then incubated with goat anti-rabbit IgG (H+L)-HRP conjugate (BIO-RAD, Hercules, CA, USA). Specific complexes were visualised with an echochemiluminescence (ECL) detection system (GE Healthcare, Little Chalfont, UK).

### Prediction of miRNA targets

Oligo-microarray human 44K (Agilent) was used for expression profiling of *miR-1-* and *miR-133a*-transfected PCa cell lines (PC3 and DU145) in comparison with *miR-negative control* transfectants as previously described (Sugimoto *et al*, 2009). In brief, hybridisation and washing steps were performed in accordance with the manufacturer's instructions. The arrays were scanned using a Packard GSI Lumonics ScanArray 4000 (Perkin-Elmer, Boston, MA, USA). The data obtained were analysed with DNASIS array software (Hitachi Software Engineering, Tokyo, Japan), which converted the signal intensity for each spot into the text format. The log2 ratios of the median subtracted background intensity were analysed. Data from each microarray study were subjected to global normalisation.

The predicted target genes and their miRNA binding site seed regions were investigated using TargetScan (release 5.1, http://www.targetscan.org/). The sequences of the predicted mature miRNAs were confirmed by referring to miRBase (release 17.0; http://microrna.sanger.ac.uk/).

### Plasmid construction and dual-luciferase reporter assays

*miR-1* and *miR-133a* target sequences were inserted between the *Xho*I–*Pme*I restriction sites in the 3′UTR of the *hRluc* gene in the psiCHECK-2 vector (C8021; Promega, Madison, WI, USA). Full-length 3′UTR sequences of *PNP* mRNA and specific miRNA target sequences (Supplementary Table 1) for *miR-1* and *miR-133a* were artificially synthesised and inserted into the vector. In addition, we constructed three mutant vectors in which the specific sites targeted by the miRNAs were deleted. Following that, PC3 cells were transfected with 5 ng of vector, 10 nM of miRNA, and 1 *μ*l of Lipofectamine 2000 (Invitrogen) in 100 *μ*l of Opti-MEM (Invitrogen). The activities of firefly and *Renilla* luciferases in cell lysates were determined with a dual-luciferase assay system (E1910; Promega). Normalised data were calculated as the quotient of *Renilla*/firefly luciferase activities.

### Immunohistochemistry

A tissue microarray containing 60 PCa specimens, 10 prostatic intraepithelial neoplasias (PINs), and 10 prostatic hyperplastic samples was obtained from Provitro (Berlin, Germany) (Cat #401 2209, Lot #146P260710.1-11, Berlin, BRD). Detailed information on all tumour specimens can be found at http://www.provitro.co.uk/Neoplastic-tumour-TMA.82.0.htmL. Immunostaining was done on the tissue microarray following the manufacturer's protocol. The primary rabbit polyclonal antibodies against PNP (Sigma-Aldrich) were diluted 1 : 450. The slides were treated with biotinylated goat anti-rabbit IgG (H+L) (Vector Laboratories, Burlingame, CA, USA). Diaminobenzidine-hydrogen peroxide (Sigma-Aldrich) was the chromogen and the counterstaining was done with 0.5% haematoxylin. Immunostaining was evaluated according to the scoring method as described previously (Zhang *et al*, 2010). Each case was scored on the basis of the intensity and area of staining. The intensity of staining was graded on the following scale: 0, no staining; 1+, mild staining; 2+, moderate staining; and 3+, intense staining. The area of staining was evaluated as follows: 0, no staining of cells in any microscopic field; 1+, <30% of cells stained positive; 2+, 30–60% stained positive; and 3+, >60% stained positive. The immunostaining scores (intensity+extent) were combined and analysed.

### Statistical analysis

The relationship between two variables and the numerical values obtained by real-time RT–PCR were analysed using the Mann–Whitney *U*-test. The relationship among three variables and the numerical values were analysed using the Bonferroni-adjusted Mann–Whitney *U*-test. Expert StatView analysis software (version 4; SAS Institute Inc., Cary, NC, USA) was used in both cases. In the comparison among three variables, a non-adjusted statistical level of significance of *P*<0.05 corresponds to a Bonferroni-adjusted level of *P*<0.0167.

## Results

### Repressed expression of *miR-1* and *miR-133a* in PCa specimens

**Figure 1:**
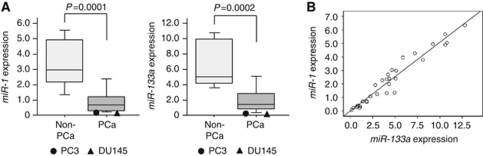
(**A**) The expression levels of *miR-1* and *miR-133a*. Real-time RT–PCR showed that the expression levels of both miRNAs were significantly lower in PCa clinical specimens than in Non-PCa specimens. Expression levels of *miR-1* and *miR-133a* in two PCa cell lines were plotted with circles as PC3 and triangles as DU145, respectively. *miR-1* and *miR-133a* expression levels were significantly lower in PC3 and DU145 cells than in non-PCa tissues. (**B**) The correlated expression of *miR-1* and *miR-133a*. The correlation coefficient of 0.571 with *P*<0.001 indicates that *miR-1* expression was highly correlated with that of *miR-133a*.

We evaluated expression levels of *miR-1* and *miR-133a* in PCa (*n*=15) and non-PCa (*n*=17) tissues. Patient characteristics are shown in Table 1 and Supplementary Table 1. RNA was extracted and miRNA expression levels of *miR-1* and *miR-133a* were determined by RT–PCR. The expression levels of *miR-1* and *miR-133a* were significantly lower in PCa compared with non-PCa tissues (*P*=0.0001 and *P*=0.0002, respectively; Figure 1A). The expression levels of *miR-1* and *miR-133a* were analysed for their correlation. A correlation coefficient of 0.571 with *P*<0.001 indicated that *miR-1* expression was highly correlated with that of *miR-133a* (Figure 1B).

### Effect of *miR-1* and *miR-133a* transfection on cell proliferation, migration, and invasive activity in PCa cell lines

We evaluated the transfection efficiency of the miRNAs in cancer cell lines based on the downregulation of mRNA expression levels of *protein tyrosine kinase 9* (*PTK9*, alias as twinfilin: *TWF1*) after *miR-1* transfection. As a result of our microarray analysis of *miR-1* transfectants, it was admitted that *PTK9* expression was downregulated (Supplementary Table 2A). This thing shows that transfection of miRNAs was effectiveness in cancer cells.

The expression levels of *miR-1* and *miR-133a* in PC3 and DU145 were determined by RT–PCR and compared with clinical specimens. The expression levels of *miR-1* and *miR-133a* were significantly lower in PC3 and DU145 than those seen in tissues (Figure 1A).

**Figure 2:**
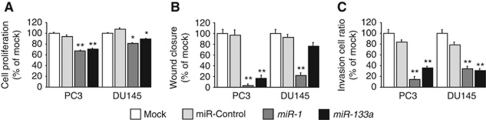
Effects of *miR-1* and *miR-133a* transfection on PC3 and DU145 cells. (**A**) Suppression of PC3 and DU145 cell proliferation after transfection with either miRNA as determined by XTT assay. (**B**) Suppression of PC3 and DU145 cell migration activity after miRNA transfection as determined by the wound-healing assay. (**C**) Suppression of PC3 and DU145 cell invasion activity after transfection with miRNAs as determined with the Matrigel invasion assay. ^**^P<0.0001; ^*^P<0.0005.

To examine the functional roles of *miR-1* and *miR-133a*, we performed gain-of-function studies using miRNA transfections into PC3 and DU145 cells. The XTT assay revealed cell proliferation inhibition in the two miRNA-transfected PCa cell lines in comparison with mock and the control transfectants (Figure 2A).

The wound-healing assay demonstrated significant cell migration inhibitions in these miRNA-transfected PC3 cell lines compared with their counterparts (Figure 2B). However, no significant inhibition was observed in *miR-133a*-transfected DU145 cell lines (Figure 2B). Supplementary Figure 2A shows the actual images of the wound-healing assay.

The Matrigel invasion assay demonstrated that the number of invading cells was significantly decreased in these miRNA transfectants compared with their counterparts (Figure 2C). Supplementary Figure 2B shows the actual images of the invasion assay.

It is plausible the *miR-1*/*miR-133a* cluster may have important role as tumour suppressors through downregulating these oncogenic genes. However, we found no simultaneous effect of cell viability inhibition by *miR-1* and *miR-133a* co-transfection (Supplementary Figure 1).

### Identification of *miR-1* and *miR-133a* target genes by genome-wide gene expression analysis

To identify the target genes of *miR-1* and *miR-133a*, we performed genome-wide gene expression analysis with *miR-1*, *miR-133a,* and negative-control miRNA transfections into PC3 and DU145 cells. Downregulated genes in either *miR-1* or *miR-133a* transfectants with less than a −1.0 (log2 ratio) downregulation compared with the control transfectants are shown in Supplementary Table 2A and B.

According to the microarray data, a total of 14 genes were downregulated by both *miR-1* and *miR-133a* transfections (Table 2). The Target Scan Program showed that six genes had putative target sites of both *miR-1* and *miR-133a* in their 3′UTR (*TAGLN2:* transgelin 2, *WDR78:* WD repeat domain 78, *C4orf34:* chromosome 4 open reading frame 34, *PNP:* purine nucleoside phosphorylase, *LASS2:* LAG1 homologue, ceramide synthase 2 and *STXBP4:* syntaxin binding protein 4; Table 2). To confirm the expression levels of these genes in the clinical specimens (PCa and non-PCa tissues), RT–PCR was performed. The data showed that *PNP* was highly expressed in the PCa specimens compared with non-PCa tissues (Supplementary Figure 4). These results indicated that *PNP* was a possible target gene of *miR-1* and *miR-133a* and function as an oncogene. The current microarray data were approved by the Gene Expression Omnibus (GEO) and were assigned GEO accession number GSE26032.

### *PNP* as a target of post-transcriptional repression by *miR-1* and *miR-133a*

**Figure 3:**
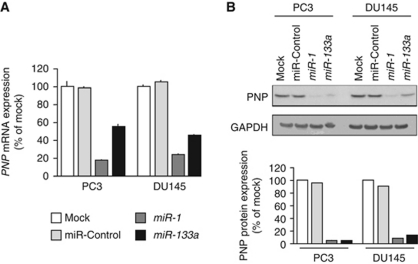
PNP expression was suppressed by *miR-1* and *miR-133a* transfection at both the mRNA and protein levels in PCa cell lines, PC3 and DU145. (**A**) *PNP* mRNA expression after 72 h transfection with *miR-1* and *miR-133a*. *GAPDH* expression was used for normalisation. (**B**) PNP protein expression after 72 h transfection of miRNAs. GAPDH was used as a loading control. The expression ratio of PNP/GAPDH was evaluated using ImageJ software (ver. 1.43; http://rsbweb.nih.gov/ij/index.htmL).

**Figure 4:**
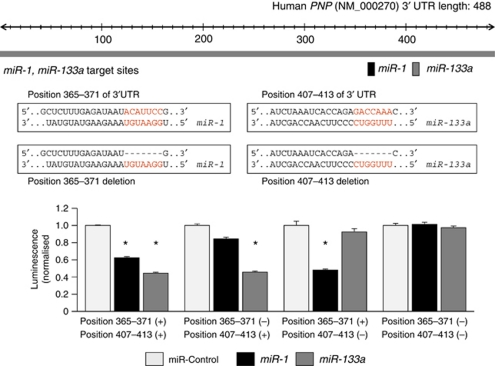
*miR-1* and *miR-133a* binding sites in 3′UTR of *PNP* mRNA. Luciferase reporter assay using the four types of vector encoding the putative *miR-1* and *miR-133a* target sites; Position 365–371(+):Position 407–413 (+), deletion of putative *miR-1* target site; Position 365–371(−):Position 407–413 (+), deletion of putative *miR-133a* target site; Position 365–371(+):Position 407–413 (−), deletion of putative target sites of both *miR-1 and miR-133a*; Position 365–371(−):Position 407–413 (−). The *Renilla* luciferase values were normalised to firefly luciferase values. ^*^P<0.01.

The mRNA and protein expression levels of PNP were markedly downregulated in *miR-1-* and *miR-133a*-transfected PC3 and DU145 cells in comparison with the mock and control transfectants (Figure 3A and B). We performed a luciferase reporter assay to determine whether *PNP* mRNA had a functional target site for *miR-1* and *miR-133a*. We used a vector encoding the 3′UTR of *PNP* mRNA including target sites for *miR-1* and *miR-133a* (positions 365–371 and 407–413, respectively; Supplementary Table 3) and found that the luminescence intensity was significantly reduced by transfectants of vector contained wild-type sequence in vector (Figure 4). The luminescence intensity significantly decreased in the presence of either sites targeted by *miR-1* or *miR-133a*, while the luminescence intensity was not decreased when the seed sequence of both target sites was deleted from the vectors (Figure 4; Supplementary Table 3). These data suggest that *miR-1* and *miR-133a* directly bind to specific sites on 3′UTR of *PNP* mRNA.

### Effect of *PNP* knockdown on cell proliferation, migration, and invasion activity in PCa cell lines

The expression levels of *PNP* mRNA were upregulated in PC3 and DU145 cells compared with non-PCa tissues (Supplementary Figure 5). There was no inverse correlation between the mRNA expression of *miR-1/miR-133a* and *PNP* in clinical specimens in this study.

**Figure 5:**
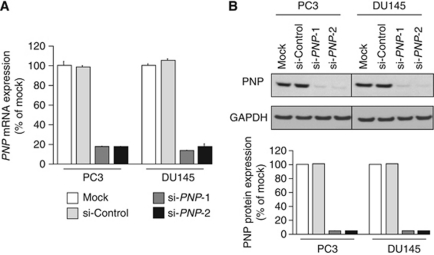
PNP expression was suppressed by *si-PNP-1 and si-PNP-2* transfection at both the mRNA and protein levels in PCa cell lines, PC3 and DU145. (**A**) *PNP* mRNA expression 72 h after transfection of the si-RNAs. *GAPDH* expression was used for normalisation. (**B**) PNP protein expression 72 h after transfection of the si-RNAs. GAPDH was used as a loading control. The expression ratio of PNP/GAPDH was evaluated using ImageJ software (ver. 1.43; http://rsbweb.nih.gov/ij/index.htmL).

To examine the functional role of PNP, we performed loss-of-function studies using two different *si-PNP* transfections into PC3 and DU145 cells. Expression of both *PNP* mRNA and PNP protein was markedly repressed in *si-PNP* transfectants (Figure 5A and B).

**Figure 6:**
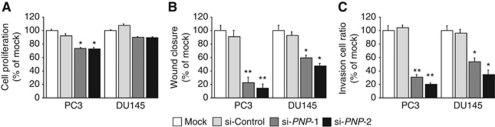
Effects of *PNP* knockdown by si-*PNP* transfection on PCa cell lines, PC3 and DU145. (**A**) Cell proliferation determined by the XTT assay; (**B**) cell migration activity determined by the wound-healing assay; and (**C**) cell invasion activity determined by the Matrigel invasion assay. ^**^P<0.0001. ^*^P<0.01.

The XTT assay revealed cell proliferation inhibition in the two *si-PNP*-transfected PC3 cell lines in comparison with those of the mock and the *si-control* transfectants (*P*<0.0001; Figure 6A). However, no significant inhibition was observed in DU145 cell lines (Figure 6A).

The wound-healing assay demonstrated significant cell migration inhibitions in the two *si-PNP* transfectants compared with their counterparts (Figure 6B). Supplementary Figure 3A shows the actual images of the wound-healing assay.

The Matrigel invasion assay demonstrated that the number of invading cells was significantly decreased in the two *si-PNP* transfectants compared with their counterparts (Figure 6C). Supplementary Figure 3B shows the actual images of the invasion assay.

### Immunohistochemistry of PNP in tissue microarray

**Figure 7:**
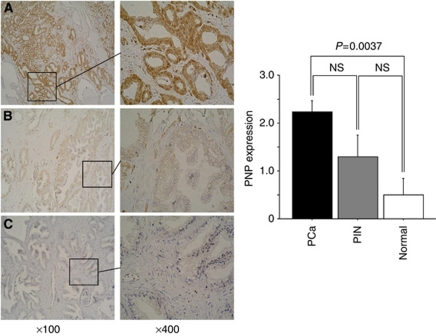
Immunohistochemical staining of PNP in PCa (*n*=60), PIN (*n*=10), and normal prostatic tissue (*n*=10) by tissue microarray (left panel, original magnification × 100; right panel, original magnification × 400). (**A**) Strongly stained tumour lesion (Gleason Score 3+4, pT3N0); (**B**) weakly stained PIN lesion; and (**C**) negative staining in hyperplastic tissue without malignancy. Right side of the figure: Quantification of PNP expression. Expression of PNP was upregulated in PCa specimens compared with normal hyperplastic tissue (*P*=0.0037).

Purine nucleoside phosphorylase was detected by immunohistochemical staining. We compared the expression levels in PCa, intraepithelial neoplasm (PIN) and normal prostatic tissue. The PNP was strongly expressed in several tumour lesions (Gleason Score 3+4, pT3aN0; Figure 7A) and PIN lesions (Figure 7B), whereas no or low expression was observed in the normal tissues (Figure 7C). The expression score of PCa was significantly higher than that of normal tissues (*P*=0.0037; Figure 7). There was no significant difference of expression of PNP analysed with clinicopathological parameters.

## Discussion

Innumerable articles have reported that miRNAs are aberrantly expressed in many types of human cancers. miRNA expression profiles of PCa also have been reported by many researchers (Porkka *et al*, 2007; Ozen *et al*, 2008; Gandellini *et al*, 2009; Schaefer *et al*, 2010). While some controversy remains, it is certain that genome-wide expression analysis contributes to our understanding of miRNA's role as oncogenes and/or tumour suppressors (Nicolas *et al*, 2011). For example, *miR-15a/16-1* that are clustered together on chromosome 13q14 are established as tumour suppressive miRNAs in human cancers including PCa (Aqeilan *et al*, 2010). Downregulation of *miR-15a* and *miR-16-1* was frequently found in PCa specimens (Porkka *et al*, 2011). The restoration of both miRNAs in LNCaP cells showed a dramatic apoptotic effect *in vitro* and *in vivo* as they targeted multiple oncogenes such as *BCL2*, *CCND1,* and *WNT3A* (Bonci *et al*, 2008). Interestingly, *BCL2* is overexpressed in the majority of patients with hormone-refractory PCa, and mediates resistance to androgen ablation and chemotherapy (Yoshino *et al*, 2006). These data strongly suggest that downregulation of *miR-15a/16-1* cluster and overexpression of BCL2 might contribute to PCa oncogenesis.

Like the *miR-15a/16-1* cluster, several other miRNAs are located in the same chromosomal region. We recognised that *miR-1* and *miR-133a* formed clusters in the human genome (18q11.2 and 20q13.33). Furthermore, our miRNA expression signatures revealed that both *miR-1* and *miR-133a* were downregulated in several types of cancer such as head and neck cancer, oesophageal cancer, and bladder cancer (Kano *et al*, 2010; Nohata *et al*, 2011a; Yoshino *et al*, 2011). In this study, we first tested the expression levels of *miR-1* and *miR-133a* using needle biopsies of PCa (*n*=15) and non-PCa (*n*=17) tissues. We confirmed reduction of both miRNAs in PCa tissues, and suggested that these changes are key steps in oncogenesis or progression in PCa. Next, we investigated the functional significance of *miR-1* and *miR-133a* in PCa cells, PC3 and DU145. Our data revealed that restoration of *miR-1* or *miR-133a* expression suppressed cancer cell proliferation, migration, and invasion in PC3 and DU145 cells. Our latest data from head and neck squamous cell carcinoma (SCC) and bladder cancer showed that *miR-1* and *miR-133a* had a role of tumour suppressors based upon functional analysis of cancer cells (Nohata *et al*, 2011b; Yoshino *et al*, 2011). As for the relationship between human cancer and *miR-1*, recent articles revealed that *miR-1* induced apoptosis through repression of *Mcl-1* in lung cancer (Nasser *et al*, 2008). *miR-1* also targets *c-Met* in rhabdomyosarcoma (Yan *et al*, 2009). With regard to *miR-133a*, several reports showed that *miR-133a* was underexpressed in pancreatic ductal adenocarcinoma, colon cancer and tongue SCC (Szafranska *et al*, 2007; Wong *et al*, 2008; Sarver *et al*, 2009). In tongue SCC, *miR-133a* inhibited cell proliferation and induced apoptosis and directly bound to oncogenic *PKM2* (Wong *et al*, 2008). Taken together, *miR-1* and *miR-133a* appear to be important miRNAs, acting as tumour suppressors in several human cancers including PCa.

miRNAs are unique in their ability to regulate many protein-coding genes. The elucidation of new networks of cancer is important for our understanding of oncogenesis. Based on this view, we continue our investigation of tumour suppressive miRNAs and their regulation of oncogenic targets in various cancer cells (Kano *et al*, 2010; Chiyomaru *et al*, 2010a, 2011; Fuse *et al*, 2011; Nohata *et al*, 2011b; Uchida *et al*, 2011; Yoshino *et al*, 2011). In this study, we adopted a method of genome-wide gene expression analysis in PC3 and DU145 cells, using *miR-1* or *miR-133a* transfectants to identify targets. Genes regulated by both *miR-1* and *miR-133a* were identified by examining expression profiles of *miR-1* and *miR-133a* transfectants.

Recently, important articles were published regarding *miR-1* and *miR-133a* target genes in rhabdomyosarcoma. The expression levels of *miR-1* and *miR-133a* were reduced in cell lines of rhabdomyosarcoma. *miR-1* and *miR-133a* regulate myogenesis by controlling distinct aspects of the differentiation process (Rao *et al*, 2006) and have important roles in the proliferation and differentiation of rhabdomyosarcoma (Rao *et al*, 2010). To investigate the contribution of two miRNAs in rhabdomyosarcoma cells, the expression signatures of *miR-1* and *miR-133a* transfectants were reported (Rao *et al*, 2010). According to the report, *miR-1* exerts a strong promyogenic influence on these poorly differentiated tumour cells. The expression signatures of rhabdomyosarcoma and our current signatures were compared. In all, 23 of 62 (37.1%) and 5 of 24 (20.8%) downregulated genes by *miR-1* and *miR-133a* transfectants in rhabdomyosarcoma were recognised in our PCa signatures, respectively. This result indicates that the downregulated genes which are common in two signatures contribute to malignant human cells. In addition, the functional analysis of *miR-1* or *miR-133a* independently regulated genes is another important theme in cancer research field.

In this study, we focused target genes directly downregulated by both *miR-1* and *miR-133a*, which contain *miR-1* and *miR-133a* binding site on their genome sequences. We investigated the mRNA expression levels of six candidate genes (*TAGLN2*, *WDR78*, *C4orf34*, *PNP*, *LASS2*, and *STXBP4*) using PCa or non-PCa clinical specimens. In this analysis, our criterion for selection was that candidate genes were upregulated in cancer tissues. One gene, *PNP*, was chosen for this standard.

To examine the role of PNP in PCa, we examined its expression by immunohistochemical analysis of tissue microarrays. Our data demonstrated that PNP was highly expressed in PCa while it was scarcely stained in normal prostatic tissues. Importantly, we noted intermediate staining of PIN tissues. Our immunohistochemical analysis indicates an important role of PNP in PCa oncogenesis. The phosphorolytic cleavage of inosine, deoxyinosine, guanosine, and deoxyguanosine to the corresponding base and sugar 1 phosphate was catalysed by PNP. Purine nucleoside phosphorylase-deficiency syndrome exhibits profound impairment in the T-cell component, in which 2′-deoxyguanosine accumulates in plasma and deoxyguanosine triphosphate (dGTP) in lymphocytes, thereby leading to dGTP-directed inhibition of DNA synthesis and cell death (Markert, 1991; Arpaia *et al*, 2000). Purine nucleoside phosphorylase is considered as a therapeutic target in malignant lymphoproliferative diseases. Forodesine (BCX-1777) is a potent inhibitor of human PNP and which induces apoptosis of chronic lymphocytic leukaemia (CLL) cells (Bantia *et al*, 2003; Ravandi and Gandhi, 2006). Phase 2 clinical trials, conducted from 2005 until 2009, used Forodesine for the treatment of patients with advanced, Fludarabine-refractory CLL (Al-Kali *et al*, 2010; Balakrishnan *et al*, 2010). Purine nucleoside phosphorylase might have functions in oncogenesis and progression of PCa; therefore, PNP inhibition might be a target of a strategy for novel treatment of PCa.

The unique point of miRNA biogenesis is that one miRNA regulates many protein-coding genes. However, the nature of miRNA–protein coding gene networks in human genome is unclear. This is why elucidation of tumour suppressive *miR-1* and *miR-133a* regulate cancer networks is important for understanding human PCa oncogenesis.

## Conclusions

*miR-1* and *miR-133a* are frequently reduced in PCa clinical specimens. Both miRNAs may function as tumour suppressors regulating *PNP,* an oncogenic gene in PCa. The *miR-1* and *miR-133a* cluster regulates novel cancer pathways and as such could provide new insights into molecular mechanisms of PCa oncogenesis and progression. Further studies could contribute to the development of new therapeutic strategies for PCa.

## Figures and Tables

**Table 1:** Patients characteristics

**Characteristic**	**Pca (*n*=15)**	**Non-Pca (*n*=17)**
*Age (years)*		
Median (range)	72 (63–88)	63 (53–85)
		
*PSA (ng ml* ^*−1*^ *)*		
Median (range)	153 (3.4–2530)	8.3 (2.8–22)
		
*T stage*		
T3a	6 (40%)	
T3b	4 (27%)	
T4	5 (33%)	
		
*N stage*		
N0	3 (20%)	
N1	12 (80%)	
		
*M stage*		
M0	7 (47%)	
M1	8 (53%)	
		
*Gleason score*		
4+3	1 (7%)	
4+4	6 (40%)	
4+5	7 (46%)	
5+5	1 (7%)	

Abbreviations: PCa=prostate cancer; PSA=prostate-specific antigen.

**Table 2:** Downregulated genes in *miR-1* and *miR-133a* transfectants

		**Fold change (log2 ratio)**						
		**PC3**		**DU145**			**Target sites**	
**Entrez Gene ID**	**Symbol**	***miR-1***	***miR-133a***	***miR-1***	***miR-133a***	**Average**	***miR-1***	***miR-133a***
8407	TAGLN2	−3.40	−2.60	−1.56	−1.71	−2.32	+	+
51776	ZAK	−1.77	−2.97	−1.38	−2.33	−2.11	−	−
79819	WDR78	−1.63	−2.91	−1.22	−1.81	−1.89	+	+
83990	BRIP1	−2.95	−1.86	−1.04	−1.68	−1.88	−	−
5819	PVRL2	−2.80	−1.57	−1.78	−1.06	−1.80	+	−
201895	C4orf34	−2.33	−2.28	−1.17	−1.38	−1.79	+	+
4860	PNP	−2.14	−1.31	−2.08	−1.54	−1.77	+	+
29956	LASS2	−1.15	−2.45	−1.12	−1.48	−1.55	+	+
79026	AHNAK	−1.33	−2.15	−1.23	−1.23	−1.48	−	−
4323	MMP14	−1.21	−1.01	−1.52	−1.54	−1.32	−	+
53340	SPA17	−1.43	−1.00	−1.42	−1.11	−1.24	−	−
252983	STXBP4	−1.03	−1.40	−1.09	−1.35	−1.22	+	+
56267	CCBL2	−1.14	−1.22	−1.14	−1.02	−1.13	−	+
146779	EFCAB3	−1.10	−1.08	−1.16	−1.17	−1.13	−	−

## Supplementary Material

Supplementary Figure Legends

Supplementary Table 2A

Supplementary Table 2B
