# Supplementary material for: Tumour suppressors miR-1 and miR-133a target the oncogenic function of purine nucleoside phosphorylase (PNP) in prostate cancer
Source: Br J Cancer. 2011 Nov 8;106(2):405–13. doi: 10.1038/bjc.2011.462 (PMC3261671; doi:10.1038/bjc.2011.462)
Supplement: Supplementary Figures 1, 4 and 5 [file bjc2011462x1.ppt]

## Slide 1
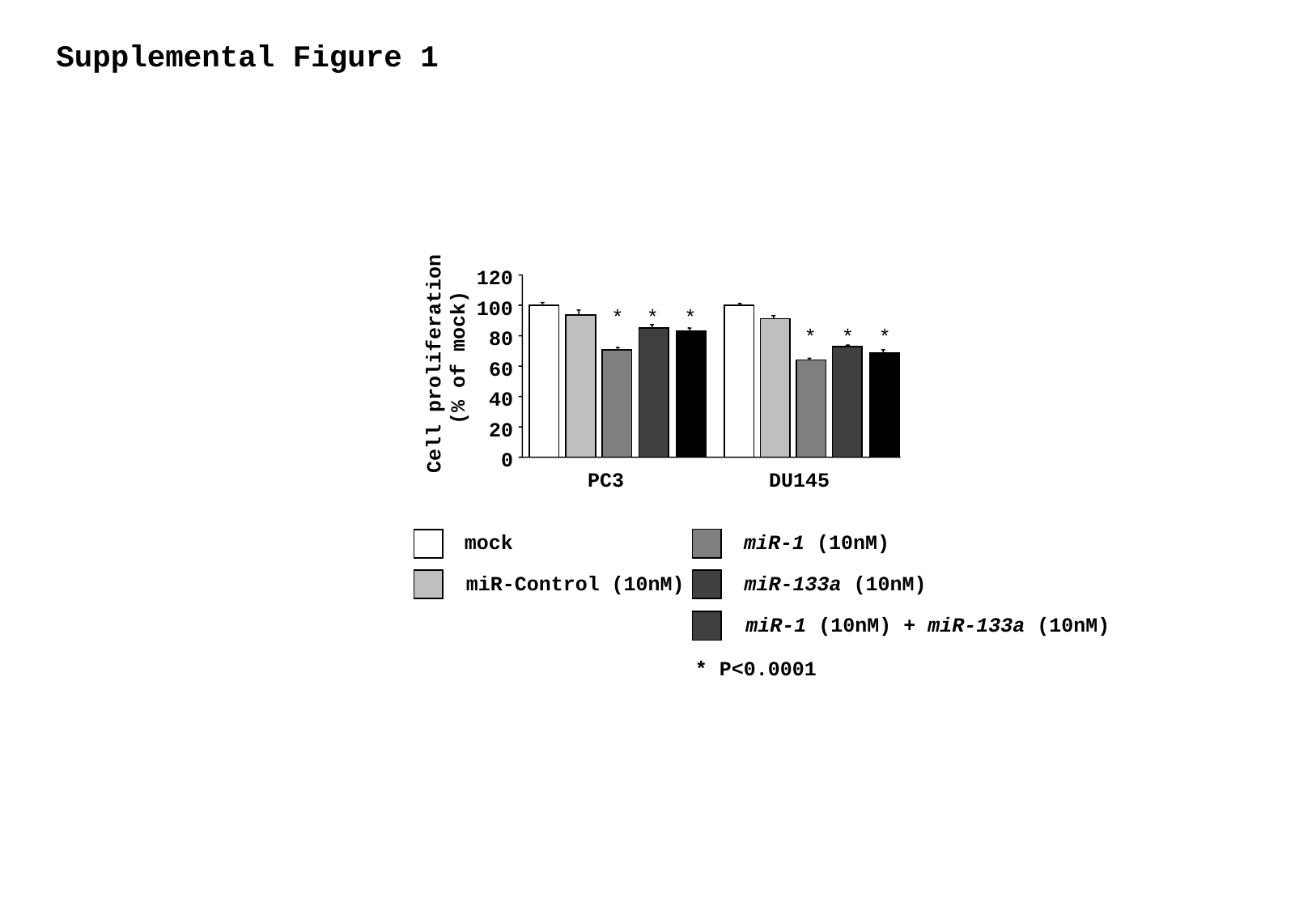

Supplemental Figure 1
120
100
*
*
*
*
*
*
80
Cell proliferation
(% of mock)
60
40
20
0
PC3
DU145
mock
miR-1 (10nM)
miR-Control (10nM)
miR-133a (10nM)
miR-1 (10nM) + miR-133a (10nM)
* P<0.0001

## Slide 2
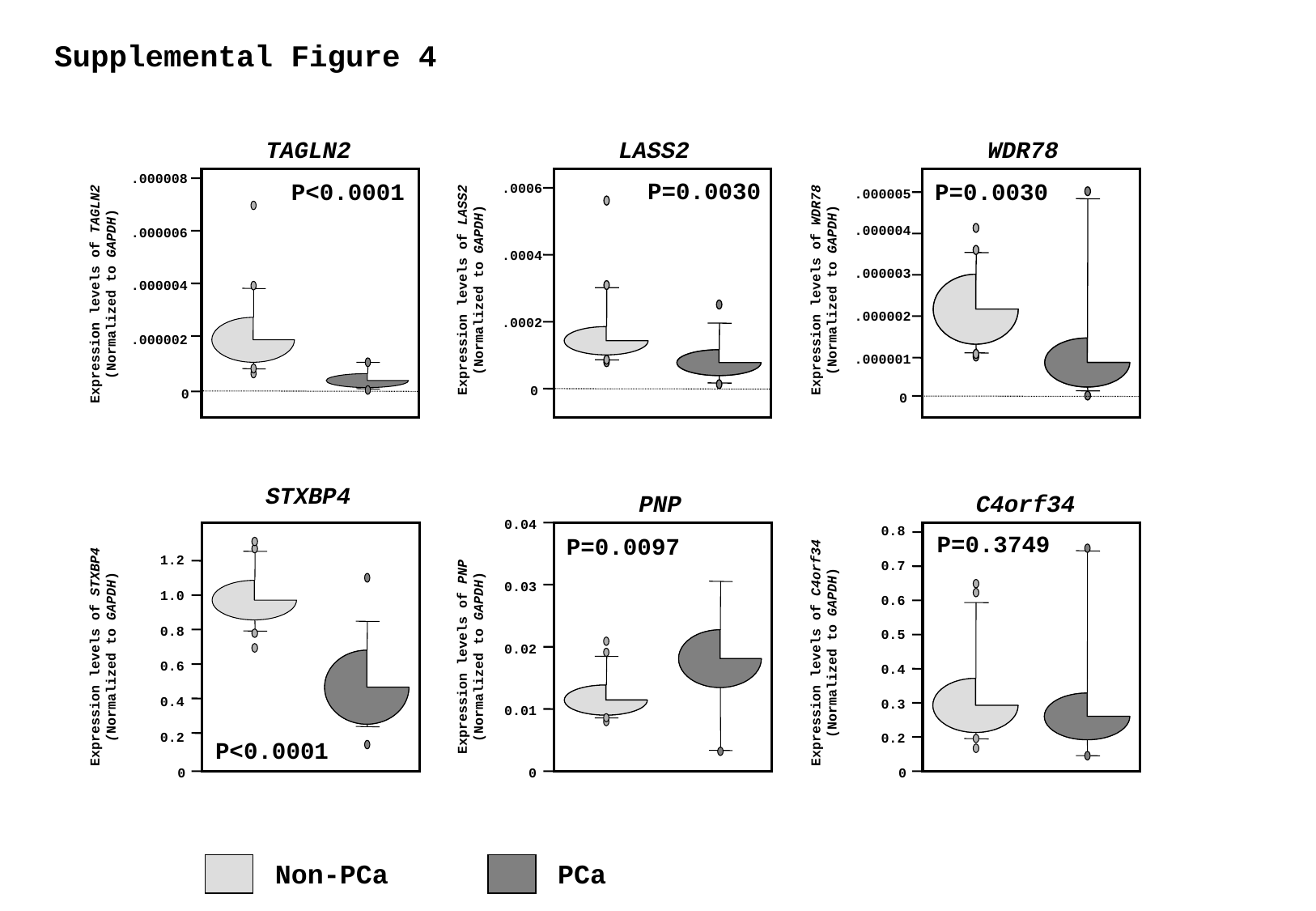

Supplemental Figure 4
TAGLN2
.000008
.000006
.000004
.000002
0
LASS2
.0006
.0004
.0002
0
WDR78
P=0.0030
P<0.0001
P=0.0030
.000005
.000004
.000003
Expression levels of LASS2
(Normalized to GAPDH)
Expression levels of WDR78
(Normalized to GAPDH)
Expression levels of TAGLN2
(Normalized to GAPDH)
.000002
.000001
0
STXBP4
1.2
1.0
0.8
0.6
0.4
0.2
0
PNP
0.04
0.03
0.02
0.01
0
C4orf34
0.8
0.7
0.6
0.5
0.4
0.3
0.2
0
P=0.3749
P=0.0097
Expression levels of C4orf34
(Normalized to GAPDH)
Expression levels of PNP
(Normalized to GAPDH)
Expression levels of STXBP4
(Normalized to GAPDH)
P<0.0001
Non-PCa
PCa

## Slide 3
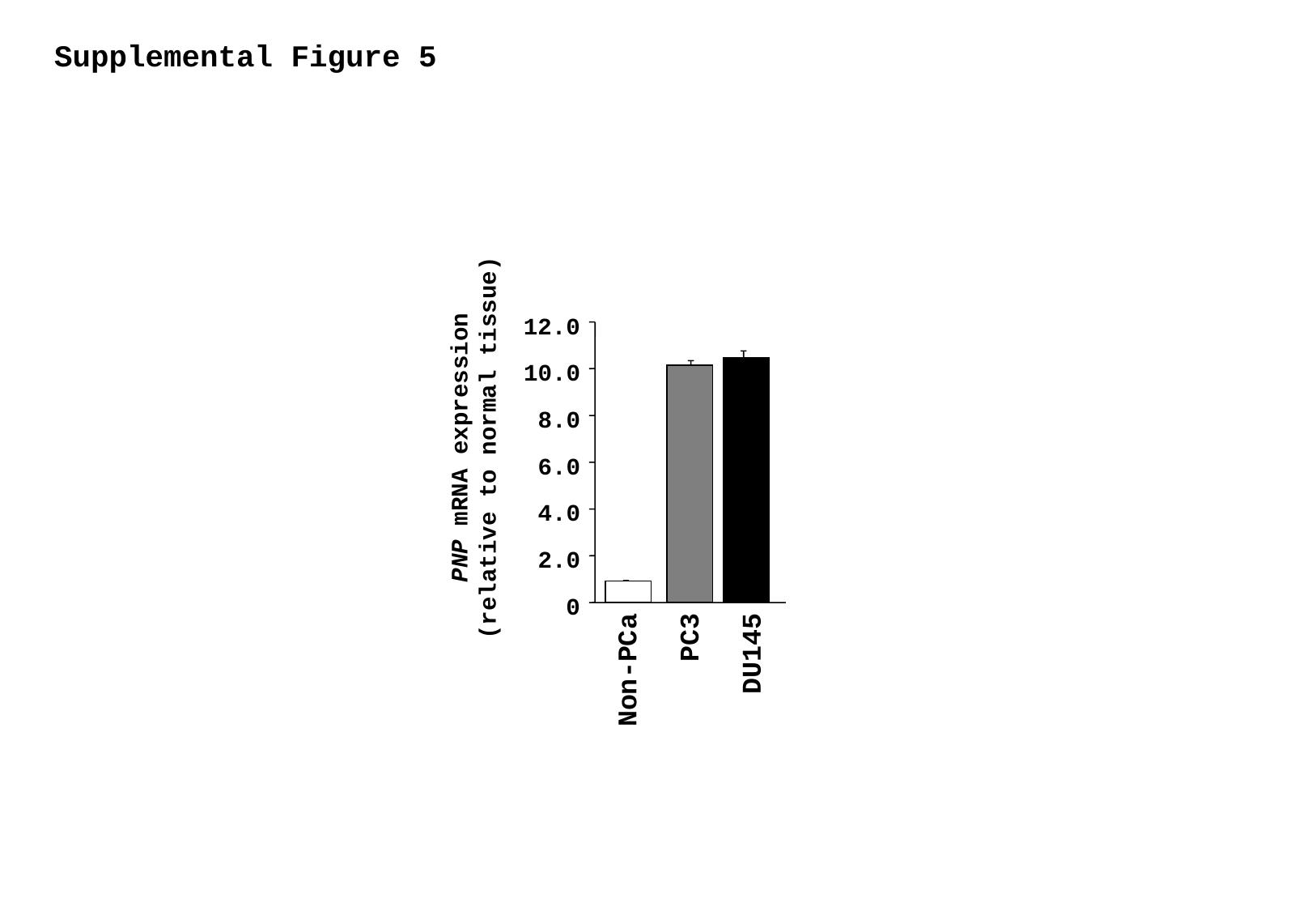

Supplemental Figure 5
12.0
10.0
8.0
PNP mRNA expression
(relative to normal tissue)
6.0
4.0
2.0
0
PC3
DU145
Non-PCa
