# Supplementary material for: Tumour suppressors miR-1 and miR-133a target the oncogenic function of purine nucleoside phosphorylase (PNP) in prostate cancer
Source: Br J Cancer. 2011 Nov 8;106(2):405–13. doi: 10.1038/bjc.2011.462 (PMC3261671; doi:10.1038/bjc.2011.462)
Supplement: Supplementary Figures 2 and 3 [file bjc2011462x2.ppt]

## Slide 1
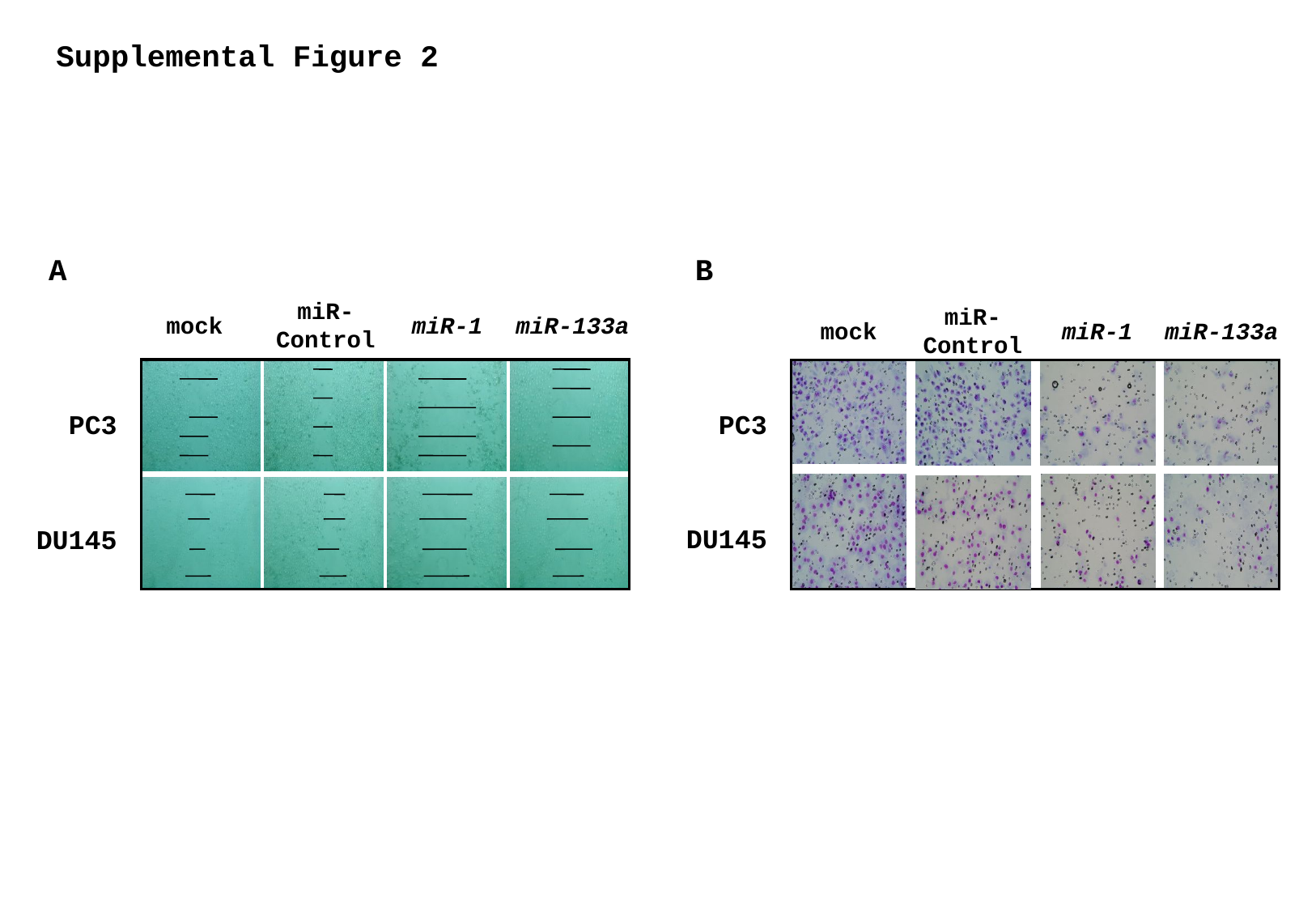

Supplemental Figure 2
A
B
miR-
Control
mock
miR-1
miR-133a
PC3
DU145
miR-
Control
mock
miR-1
miR-133a
PC3
DU145

## Slide 2
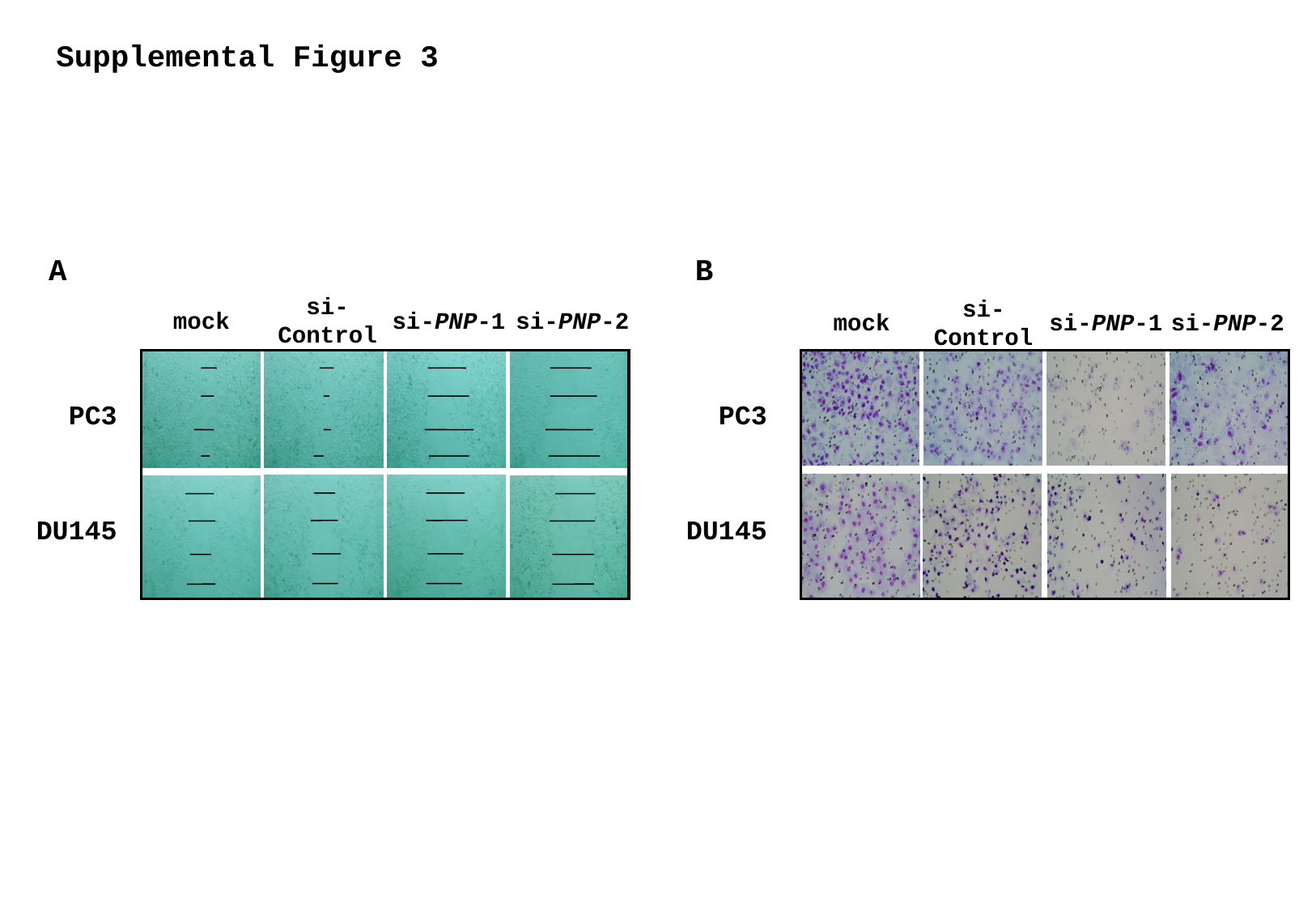

Supplemental Figure 3
A
B
si-
Control
si-PNP-1
si-PNP-2
mock
PC3
DU145
si-
Control
si-PNP-1
si-PNP-2
mock
PC3
DU145
