# Supplementary material for: Tumour suppressors miR-1 and miR-133a target the oncogenic function of purine nucleoside phosphorylase (PNP) in prostate cancer
Source: Br J Cancer. 2011 Nov 8;106(2):405–13. doi: 10.1038/bjc.2011.462 (PMC3261671; doi:10.1038/bjc.2011.462)
Supplement: Supplementary Figure Legends [file bjc2011462x3.doc]

**Supplemental Figure 1**

Effect of *miR-1* and *miR-133a* co-transfection on PC3 and DU145 cells.

Suppression of PC3 and DU145 cell proliferation after transfection with *miR-1*, *miR-133a* and co-transfection of *miR-1/miR-133a* as determined by XTT assay. **P* < 0.0001

**Supplemental Figure 2**

Effects of *miR-1* and *miR-133a* transfection on PC3 and DU145 cells.

(**A**) cell migration activity determined by the wound healing assay. (**B**) cell invasion activity determined by the Matrigel invasion assay.

**Supplemental Figure 3**

Effects of *PNP*-knockdown by si-*PNP* transfection on PC3 and DU145 cells.

(**A**) cell migration activity determined by the wound healing assay. (**B**) cell invasion activity determined by the Matrigel invasion assay.

**Supplemental Figure 4**

mRNA expression levels of six candidate genes in PCa clinical specimens.

Expression levels of six genes (*TAGLN2*, *LASS2*, *WDR78*, *STXBP4*, *PNP* and *C4orf34*) was evaluated by Non-PCa tissues (n = 17) and PCa (n = 15). Real-time RT-PCR showed that expression level of *PNP* mRNA in PCa tissues were significantly higher level than in the Non-PCa. *GAPDH* was used as internal control. TaqMan probes and primers for *TAGLN2* (Hs00761239_s1), *LASS2* (Hs00371958_g1), *WDR78* (Hs00227012_m1), *STXBP4* (Hs00736692_m1), *PNP* (Hs00165367_m1) and *C4orf34* (Hs00383605_m1) were obtained from Applied Biosystems (Assay-On-Demand Gene Expression Products).

**Supplemental Figure 5**

mRNA expression level of *PNP* in PC3 and DU145 cells.

*PNP* mRNA expression was significantly increased in PC3 and DU145 cells compared to Non-PCa tissues. *GAPDH* expression was used for normalization.
