# Supplementary material for: Tumour suppressors miR-1 and miR-133a target the oncogenic function of purine nucleoside phosphorylase (PNP) in prostate cancer
Source: Br J Cancer. 2011 Nov 8;106(2):405–13. doi: 10.1038/bjc.2011.462 (PMC3261671; doi:10.1038/bjc.2011.462)
Supplement: Supplementary Table 2A [file bjc2011462x4.doc]

**Supplemenatal table 2A**

Down-regulated genes by in miR-1-transfected PC3 and DU145 cells in comparison with the control

| Entrez Gene ID | Gene symbol | Gene name | Log2 ratio (PC3) | Log2 ratio (DU145) | Log2 ratio (avarage) |
| --- | --- | --- | --- | --- | --- |
| 23446 | SLC44A1 | solute carrier family 44, member 1 | -3.38 | -2.33 | -2.86 |
| 27230 | SERP1 | stress-associated endoplasmic reticulum protein 1 | -2.96 | -2.56 | -2.76 |
| 8407 | TAGLN2 | transgelin 2 | -3.40 | -1.56 | -2.48 |
| 23531 | MMD | monocyte to macrophage differentiation-associated | -3.07 | -1.84 | -2.46 |
| 79794 | C12orf49 | chromosome 12 open reading frame 49 | -2.90 | -1.88 | -2.39 |
| 2697 | GJA1 | gap junction protein, alpha 1, 43kDa | -2.70 | -2.02 | -2.36 |
| 5819 | PVRL2 | poliovirus receptor-related 2 (herpesvirus entry mediator B) | -2.80 | -1.78 | -2.29 |
| 5756 | TWF1 | twinfilin, actin-binding protein, homolog 1 (Drosophila) | -2.33 | -2.15 | -2.24 |
| 359845 | FAM101B | family with sequence similarity 101, member B | -2.62 | -1.73 | -2.17 |
| 4860 | PNP | purine nucleoside phosphorylase | -2.14 | -2.08 | -2.11 |
| 1906 | EDN1 | endothelin 1 | -2.19 | -1.99 | -2.09 |
| 84650 | EBPL | emopamil binding protein-like | -2.41 | -1.74 | -2.08 |
| 55916 | NXT2 | nuclear transport factor 2-like export factor 2 | -2.48 | -1.65 | -2.06 |
| 54502 | RBM47 | RNA binding motif protein 47 | -2.76 | -1.36 | -2.06 |
| 81831 | NETO2 | neuropilin (NRP) and tolloid (TLL)-like 2 | -2.38 | -1.73 | -2.06 |
| 83990 | BRIP1 | BRCA1 interacting protein C-terminal helicase 1 | -2.95 | -1.04 | -2.00 |
| 84650 | EBPL | emopamil binding protein-like | -2.16 | -1.73 | -1.94 |
| 8544 | PIR | pirin (iron-binding nuclear protein) | -2.18 | -1.68 | -1.93 |
| 84747 | UNC119B | unc-119 homolog B (C. elegans) | -1.96 | -1.90 | -1.93 |
| 10971 | YWHAQ | tyrosine 3-monooxygenase/tryptophan 5-monooxygenase activation protein, theta polypeptide | -2.73 | -1.04 | -1.89 |
| 89765 | RSPH1 | radial spoke head 1 homolog (Chlamydomonas) | -1.84 | -1.88 | -1.86 |
| 352909 | C19orf51 | chromosome 19 open reading frame 51 | -1.53 | -2.15 | -1.84 |
| 5757 | PTMA | prothymosin, alpha | -2.04 | -1.64 | -1.84 |
| 3021 | H3F3B | H3 histone, family 3B (H3.3B) | -1.88 | -1.78 | -1.83 |
| 374897 | SBSN | suprabasin | -1.99 | -1.66 | -1.83 |
| 3589 | IL11 | interleukin 11 | -2.27 | -1.34 | -1.80 |
| 79850 | FAM57A | family with sequence similarity 57, member A | -2.05 | -1.52 | -1.79 |
| 57542 | KLHDC5 | kelch domain containing 5 | -1.88 | -1.67 | -1.78 |
| 201895 | C4orf34 | chromosome 4 open reading frame 34 | -2.33 | -1.17 | -1.75 |
| 7295 | TXN | thioredoxin | -2.24 | -1.25 | -1.75 |
| 5311 | PKD2 | polycystic kidney disease 2 (autosomal dominant) | -2.22 | -1.26 | -1.74 |
| 51125 | GOLGA7 | golgin A7 | -1.99 | -1.45 | -1.72 |
| 23094 | SIPA1L3 | signal-induced proliferation-associated 1 like 3 | -1.96 | -1.44 | -1.70 |
| 4233 | MET | met proto-oncogene (hepatocyte growth factor receptor) | -1.72 | -1.67 | -1.70 |
| 377 | ARF3 | ADP-ribosylation factor 3 | -2.18 | -1.21 | -1.69 |
| 8683 | SFRS9 | splicing factor, arginine/serine-rich 9 | -1.71 | -1.66 | -1.69 |
| 130340 | AP1S3 | adaptor-related protein complex 1, sigma 3 subunit | -1.62 | -1.74 | -1.68 |
| 1174 | AP1S1 | adaptor-related protein complex 1, sigma 1 subunit | -1.34 | -2.00 | -1.67 |
| 27250 | PDCD4 | programmed cell death 4 (neoplastic transformation inhibitor) | -1.39 | -1.94 | -1.67 |
| 3556 | IL1RAP | interleukin 1 receptor accessory protein | -1.38 | -1.94 | -1.66 |
| 303 | ANXA2P1 | annexin A2 pseudogene 1 | -2.02 | -1.27 | -1.64 |
| 7117 | TMSL3 | thymosin-like 3 | -1.89 | -1.37 | -1.63 |
| 64858 | DCLRE1B | DNA cross-link repair 1B (PSO2 homolog, S. cerevisiae) | -1.99 | -1.27 | -1.63 |
| 27436 | EML4 | echinoderm microtubule associated protein like 4 | -1.90 | -1.36 | -1.63 |
| 6385 | SDC4 | syndecan 4 | -2.12 | -1.05 | -1.59 |
| 158584 | FAAH2 | fatty acid amide hydrolase 2 | -1.72 | -1.45 | -1.58 |
| 5796 | PTPRK | protein tyrosine phosphatase, receptor type, K | -1.99 | -1.16 | -1.58 |
| 3150 | HMGN1 | high-mobility group nucleosome binding domain 1 | -1.95 | -1.20 | -1.58 |
| 51776 | ZAK | sterile alpha motif and leucine zipper containing kinase AZK | -1.77 | -1.38 | -1.58 |
| 55276 | PGM2 | phosphoglucomutase 2 | -1.90 | -1.24 | -1.57 |
| 57645 | POGK | pogo transposable element with KRAB domain | -1.69 | -1.43 | -1.56 |
| 7170 | TPM3 | tropomyosin 3 | -1.87 | -1.24 | -1.56 |
| 51317 | PHF21A | PHD finger protein 21A | -1.58 | -1.53 | -1.56 |
| 220988 | HNRNPA3 | heterogeneous nuclear ribonucleoprotein A3 | -1.85 | -1.24 | -1.55 |
| 8309 | ACOX2 | acyl-Coenzyme A oxidase 2, branched chain | -1.23 | -1.86 | -1.54 |
| 89795 | NAV3 | neuron navigator 3 | -1.83 | -1.24 | -1.53 |
| 2764 | GMFB | glia maturation factor, beta | -1.69 | -1.37 | -1.53 |
| 65124 | ANKRD57 | ankyrin repeat domain 57 | -1.84 | -1.21 | -1.52 |
| 26578 | OSTF1 | osteoclast stimulating factor 1 | -1.90 | -1.10 | -1.50 |
| 23507 | LRRC8B | leucine rich repeat containing 8 family, member B | -1.46 | -1.55 | -1.50 |
| 7114 | TMSB4X | thymosin beta 4, X-linked | -1.70 | -1.29 | -1.50 |
| 305 | ANXA2P3 | annexin A2 pseudogene 3 | -1.89 | -1.10 | -1.50 |
| 388650 | FAM69A | family with sequence similarity 69, member A | -1.69 | -1.29 | -1.49 |
| 55754 | TMEM30A | transmembrane protein 30A | -1.80 | -1.18 | -1.49 |
| 51495 | PTPLAD1 | protein tyrosine phosphatase-like A domain containing 1 | -1.75 | -1.22 | -1.49 |
| 7852 | CXCR4 | chemokine (C-X-C motif) receptor 4 | -1.23 | -1.73 | -1.48 |
| 80195 | C10orf57 | chromosome 10 open reading frame 57 | -1.42 | -1.54 | -1.48 |
| 302 | ANXA2 | annexin A2 | -1.92 | -1.02 | -1.47 |
| 222008 | VSTM2A | V-set and transmembrane domain containing 2A | -1.62 | -1.32 | -1.47 |
| 84057 | MND1 | meiotic nuclear divisions 1 homolog (S. cerevisiae) | -1.62 | -1.30 | -1.46 |
| 10622 | POLR3G | polymerase (RNA) III (DNA directed) polypeptide G (32kD) | -1.17 | -1.75 | -1.46 |
| 1075 | CTSC | cathepsin C | -1.80 | -1.12 | -1.46 |
| 23729 | SHPK | sedoheptulokinase | -1.64 | -1.27 | -1.45 |
| 10228 | STX6 | syntaxin 6 | -1.38 | -1.53 | -1.45 |
| 200879 | LIPH | lipase, member H | -1.59 | -1.31 | -1.45 |
| 586 | BCAT1 | branched chain aminotransferase 1, cytosolic | -1.63 | -1.27 | -1.45 |
| 152006 | RNF38 | ring finger protein 38 | -1.44 | -1.45 | -1.44 |
| 81611 | ANP32E | acidic (leucine-rich) nuclear phosphoprotein 32 family, member E | -1.61 | -1.27 | -1.44 |
| 79819 | WDR78 | WD repeat domain 78 | -1.63 | -1.22 | -1.43 |
| 55775 | TDP1 | tyrosyl-DNA phosphodiesterase 1 | -1.64 | -1.22 | -1.43 |
| 53340 | SPA17 | sperm autoantigenic protein 17 | -1.43 | -1.42 | -1.43 |
| 56882 | CDC42SE1 | CDC42 small effector 1 | -1.73 | -1.12 | -1.42 |
| 9120 | SLC16A6 | solute carrier family 16, member 6 (monocarboxylic acid transporter 7) | -1.82 | -1.02 | -1.42 |
| 55776 | C6orf64 | chromosome 6 open reading frame 64 | -1.80 | -1.04 | -1.42 |
| 3918 | LAMC2 | laminin, gamma 2 | -1.49 | -1.33 | -1.41 |
| 378 | ARF4 | ADP-ribosylation factor 4 | -1.53 | -1.28 | -1.41 |
| 10487 | CAP1 | CAP, adenylate cyclase-associated protein 1 (yeast) | -1.72 | -1.09 | -1.40 |
| 113612 | CYP2U1 | cytochrome P450, family 2, subfamily U, polypeptide 1 | -1.80 | -1.01 | -1.40 |
| 22856 | CHSY1 | chondroitin sulfate synthase 1 | -1.45 | -1.36 | -1.40 |
| 22837 | COBLL1 | COBL-like 1 | -1.41 | -1.38 | -1.40 |
| 25796 | PGLS | 6-phosphogluconolactonase | -1.53 | -1.26 | -1.40 |
| 201299 | RDM1 | RAD52 motif 1 | -1.34 | -1.44 | -1.39 |
| 126731 | C1orf96 | chromosome 1 open reading frame 96 | -1.44 | -1.34 | -1.39 |
| 3667 | IRS1 | insulin receptor substrate 1 | -1.47 | -1.30 | -1.39 |
| 157769 | FAM91A1 | family with sequence similarity 91, member A1 | -1.32 | -1.44 | -1.38 |
| 650157 | LOC650157 | similar to TRIM5/CypA fusion protein | -1.63 | -1.12 | -1.37 |
| 54762 | GRAMD1C | GRAM domain containing 1C | -1.60 | -1.14 | -1.37 |
| 54964 | C1orf56 | chromosome 1 open reading frame 56 | -1.71 | -1.03 | -1.37 |
| 2531 | KDSR | 3-ketodihydrosphingosine reductase | -1.29 | -1.46 | -1.37 |
| 51497 | TH1L | TH1-like (Drosophila) | -1.44 | -1.30 | -1.37 |
| 4323 | MMP14 | matrix metallopeptidase 14 (membrane-inserted) | -1.21 | -1.52 | -1.36 |
| 10395 | DLC1 | deleted in liver cancer 1 | -1.51 | -1.20 | -1.35 |
| 8829 | NRP1 | neuropilin 1 | -1.54 | -1.16 | -1.35 |
| 4919 | ROR1 | receptor tyrosine kinase-like orphan receptor 1 | -1.43 | -1.27 | -1.35 |
| 5932 | RBBP8 | retinoblastoma binding protein 8 | -1.57 | -1.13 | -1.35 |
| 3329 | HSPD1 | heat shock 60kDa protein 1 (chaperonin) | -1.24 | -1.43 | -1.34 |
| 55032 | SLC35A5 | solute carrier family 35, member A5 | -1.51 | -1.15 | -1.33 |
| 26751 | SH3YL1 | SH3 domain containing, Ysc84-like 1 (S. cerevisiae) | -1.55 | -1.11 | -1.33 |
| 6745 | SSR1 | signal sequence receptor, alpha | -1.60 | -1.06 | -1.33 |
| 1956 | EGFR | epidermal growth factor receptor (erythroblastic leukemia viral (v-erb-b) oncogene homolog, avian) | -1.35 | -1.28 | -1.31 |
| 4201 | MEA1 | male-enhanced antigen 1 | -1.58 | -1.04 | -1.31 |
| 51175 | TUBE1 | tubulin, epsilon 1 | -1.10 | -1.52 | -1.31 |
| 7035 | TFPI | tissue factor pathway inhibitor (lipoprotein-associated coagulation inhibitor) | -1.59 | -1.03 | -1.31 |
| 55215 | FANCI | Fanconi anemia, complementation group I | -1.41 | -1.20 | -1.31 |
| 132789 | GNPDA2 | glucosamine-6-phosphate deaminase 2 | -1.12 | -1.50 | -1.31 |
| 23421 | ITGB3BP | integrin beta 3 binding protein (beta3-endonexin) | -1.46 | -1.14 | -1.30 |
| 79158 | GNPTAB | N-acetylglucosamine-1-phosphate transferase, alpha and beta subunits | -1.56 | -1.04 | -1.30 |
| 122769 | PPIL5 | peptidylprolyl isomerase (cyclophilin)-like 5 | -1.49 | -1.10 | -1.30 |
| 1363 | CPE | carboxypeptidase E | -1.57 | -1.02 | -1.30 |
| 83641 | FAM107B | family with sequence similarity 107, member B | -1.56 | -1.03 | -1.29 |
| 57600 | FNIP2 | folliculin interacting protein 2 | -1.39 | -1.20 | -1.29 |
| 79026 | AHNAK | AHNAK nucleoprotein | -1.33 | -1.23 | -1.28 |
| 164022 | PPIAL4A | peptidylprolyl isomerase A (cyclophilin A)-like 4A | -1.53 | -1.02 | -1.27 |
| 60681 | FKBP10 | FK506 binding protein 10, 65 kDa | -1.31 | -1.23 | -1.27 |
| 103 | ADAR | adenosine deaminase, RNA-specific | -1.44 | -1.09 | -1.26 |
| 5042 | PABPC3 | poly(A) binding protein, cytoplasmic 3 | -1.09 | -1.43 | -1.26 |
| 5768 | QSOX1 | quiescin Q6 sulfhydryl oxidase 1 | -1.25 | -1.27 | -1.26 |
| 10762 | NUP50 | nucleoporin 50kDa | -1.40 | -1.12 | -1.26 |
| 55179 | FAIM | Fas apoptotic inhibitory molecule | -1.34 | -1.17 | -1.26 |
| 5478 | PPIA | peptidylprolyl isomerase A (cyclophilin A) | -1.46 | -1.04 | -1.25 |
| 4580 | MTX1 | metaxin 1 | -1.28 | -1.22 | -1.25 |
| 9014 | TAF1B | TATA box binding protein (TBP)-associated factor, RNA polymerase I, B, 63kDa | -1.35 | -1.15 | -1.25 |
| 402644 | tcag7.873 | hypothetical LOC402644 | -1.48 | -1.02 | -1.25 |
| 10741 | RBBP9 | retinoblastoma binding protein 9 | -1.47 | -1.02 | -1.24 |
| 6546 | SLC8A1 | solute carrier family 8 (sodium/calcium exchanger), member 1 | -1.06 | -1.43 | -1.24 |
| 4673 | NAP1L1 | nucleosome assembly protein 1-like 1 | -1.47 | -1.02 | -1.24 |
| 11147 | HHLA3 | HERV-H LTR-associating 3 | -1.45 | -1.02 | -1.24 |
| 3486 | IGFBP3 | insulin-like growth factor binding protein 3 | -1.45 | -1.02 | -1.24 |
| 151194 | FAM119A | family with sequence similarity 119, member A | -1.16 | -1.29 | -1.23 |
| 1303 | COL12A1 | collagen, type XII, alpha 1 | -1.35 | -1.09 | -1.22 |
| 27085 | MTBP | Mdm2, transformed 3T3 cell double minute 2, p53 binding protein (mouse) binding protein, 104kDa | -1.23 | -1.21 | -1.22 |
| 54467 | ANKIB1 | ankyrin repeat and IBR domain containing 1 | -1.22 | -1.21 | -1.22 |
| 9640 | ZNF592 | zinc finger protein 592 | -1.38 | -1.06 | -1.22 |
| 65062 | ALS2CR4 | amyotrophic lateral sclerosis 2 (juvenile) chromosome region, candidate 4 | -1.31 | -1.12 | -1.22 |
| 23370 | ARHGEF18 | Rho/Rac guanine nucleotide exchange factor (GEF) 18 | -1.38 | -1.05 | -1.22 |
| 55691 | FRMD4A | FERM domain containing 4A | -1.41 | -1.02 | -1.22 |
| 55568 | GALNT10 | UDP-N-acetyl-alpha-D-galactosamine:polypeptide N-acetylgalactosaminyltransferase 10 (GalNAc-T10) | -1.23 | -1.19 | -1.21 |
| 1122 | CHML | choroideremia-like (Rab escort protein 2) | -1.25 | -1.16 | -1.20 |
| 23167 | EFR3A | EFR3 homolog A (S. cerevisiae) | -1.37 | -1.03 | -1.20 |
| 376940 | ZC3H6 | zinc finger CCCH-type containing 6 | -1.08 | -1.32 | -1.20 |
| 23023 | TMCC1 | transmembrane and coiled-coil domain family 1 | -1.21 | -1.19 | -1.20 |
| 84938 | ATG4C | ATG4 autophagy related 4 homolog C (S. cerevisiae) | -1.10 | -1.29 | -1.20 |
| 89781 | HPS4 | Hermansky-Pudlak syndrome 4 | -1.17 | -1.22 | -1.19 |
| 2820 | GPD2 | glycerol-3-phosphate dehydrogenase 2 (mitochondrial) | -1.07 | -1.30 | -1.19 |
| 286097 | EFHA2 | EF-hand domain family, member A2 | -1.16 | -1.20 | -1.18 |
| 7106 | TSPAN4 | tetraspanin 4 | -1.14 | -1.21 | -1.18 |
| 200844 | C3orf67 | chromosome 3 open reading frame 67 | -1.13 | -1.22 | -1.17 |
| 59284 | CACNG7 | calcium channel, voltage-dependent, gamma subunit 7 | -1.11 | -1.24 | -1.17 |
| 26986 | PABPC1 | poly(A) binding protein, cytoplasmic 1 | -1.08 | -1.27 | -1.17 |
| 51444 | RNF138 | ring finger protein 138 | -1.04 | -1.30 | -1.17 |
| 4214 | MAP3K1 | mitogen-activated protein kinase kinase kinase 1 | -1.24 | -1.09 | -1.17 |
| 3075 | CFH | complement factor H | -1.22 | -1.11 | -1.16 |
| 84327 | ZBED3 | zinc finger, BED-type containing 3 | -1.23 | -1.07 | -1.15 |
| 202781 | LOC202781 | hypothetical LOC202781 | -1.03 | -1.28 | -1.15 |
| 3927 | LASP1 | LIM and SH3 protein 1 | -1.17 | -1.12 | -1.15 |
| 56267 | CCBL2 | cysteine conjugate-beta lyase 2 | -1.14 | -1.14 | -1.14 |
| 3212 | HOXB2 | homeobox B2 | -1.09 | -1.19 | -1.14 |
| 29956 | LASS2 | LAG1 homolog, ceramide synthase 2 | -1.15 | -1.12 | -1.14 |
| 389722 | RP11-138L21.1 | similar to cell recognition molecule CASPR3 | -1.09 | -1.17 | -1.13 |
| 146779 | EFCAB3 | EF-hand calcium binding domain 3 | -1.10 | -1.16 | -1.13 |
| 57082 | CASC5 | cancer susceptibility candidate 5 | -1.23 | -1.04 | -1.13 |
| 80326 | WNT10A | wingless-type MMTV integration site family, member 10A | -1.16 | -1.09 | -1.13 |
| 10541 | ANP32B | acidic (leucine-rich) nuclear phosphoprotein 32 family,  member B | -1.08 | -1.17 | -1.12 |
| 55920 | RCC2 | regulator of chromosome condensation 2 | -1.10 | -1.15 | -1.12 |
| 54462 | FAM190B | family with sequence similarity 190, member B | -1.04 | -1.20 | -1.12 |
| 55604 | LRRC16A | leucine rich repeat containing 16A | -1.06 | -1.16 | -1.11 |
| 84698 | CAPS2 | calcyphosine 2 | -1.16 | -1.06 | -1.11 |
| 5479 | PPIB | peptidylprolyl isomerase B (cyclophilin B) | -1.05 | -1.17 | -1.11 |
| 170679 | PSORS1C1 | psoriasis susceptibility 1 candidate 1 | -1.01 | -1.15 | -1.08 |
| 23214 | XPO6 | exportin 6 | -1.10 | -1.05 | -1.08 |
| 64857 | PLEKHG2 | pleckstrin homology domain containing, family G (with RhoGef domain) member 2 | -1.07 | -1.07 | -1.07 |
| 6598 | SMARCB1 | SWI/SNF related, matrix associated, actin dependent regulator of chromatin, subfamily b, member 1 | -1.12 | -1.02 | -1.07 |
| 50862 | RNF141 | ring finger protein 141 | -1.10 | -1.04 | -1.07 |
| 221443 | C6orf130 | chromosome 6 open reading frame 130 | -1.10 | -1.04 | -1.07 |
| 80351 | TNKS2 | tankyrase, TRF1-interacting ankyrin-related ADP-ribose polymerase 2 | -1.02 | -1.11 | -1.06 |
| 10002 | NR2E3 | nuclear receptor subfamily 2, group E, member 3 | -1.00 | -1.12 | -1.06 |
| 252983 | STXBP4 | syntaxin binding protein 4 | -1.03 | -1.09 | -1.06 |
| 57291 | KIAA0114 | KIAA0114 | -1.02 | -1.05 | -1.04 |
| 401585 | FLJ25917 | hypothetical gene supported by AK098783 | -1.02 | -1.01 | -1.01 |
