# Supplementary material for: Tumour suppressors miR-1 and miR-133a target the oncogenic function of purine nucleoside phosphorylase (PNP) in prostate cancer
Source: Br J Cancer. 2011 Nov 8;106(2):405–13. doi: 10.1038/bjc.2011.462 (PMC3261671; doi:10.1038/bjc.2011.462)
Supplement: Supplementary Table 2B [file bjc2011462x5.doc]

**Supplemenatal table 2B**

Down-regulated genes in miR-133a-transfected PC3 and DU145 cells in comparison with the control

| Entrez Gene ID | Gene symbol | Gene name | Log2 ratio (PC3) | Log2 ratio (DU145) | Log2 ratio (avarage) |
| --- | --- | --- | --- | --- | --- |
| 51776 | ZAK | sterile alpha motif and leucine zipper containing kinase AZK | -2.97 | -2.33 | -2.65 |
| 23204 | ARL6IP1 | ADP-ribosylation factor-like 6 interacting protein 1 | -2.88 | -1.88 | -2.38 |
| 79819 | WDR78 | WD repeat domain 78 | -2.91 | -1.81 | -2.36 |
| 10092 | ARPC5 | actin related protein 2/3 complex, subunit 5, 16kDa | -2.46 | -1.85 | -2.16 |
| 8407 | TAGLN2 | transgelin 2 | -2.60 | -1.71 | -2.15 |
| 1515 | CTSL2 | cathepsin L2 | -3.00 | -1.28 | -2.14 |
| 25907 | TMEM158 | transmembrane protein 158 (gene/pseudogene) | -2.62 | -1.57 | -2.10 |
| 255758 | TCTEX1D2 | Tctex1 domain containing 2 | -2.37 | -1.73 | -2.05 |
| 2950 | GSTP1 | glutathione S-transferase pi 1 | -2.59 | -1.46 | -2.03 |
| 80071 | CCDC15 | coiled-coil domain containing 15 | -1.76 | -2.19 | -1.97 |
| 29956 | LASS2 | LAG1 homolog, ceramide synthase 2 | -2.45 | -1.48 | -1.96 |
| 1500 | CTNND1 | catenin (cadherin-associated protein), delta 1 | -2.48 | -1.38 | -1.93 |
| 80127 | C14orf45 | chromosome 14 open reading frame 45 | -2.14 | -1.71 | -1.93 |
| 55076 | TMEM45A | transmembrane protein 45A | -1.25 | -2.56 | -1.90 |
| 27166 | PRELID1 | PRELI domain containing 1 | -2.46 | -1.24 | -1.85 |
| 29843 | SENP1 | SUMO1/sentrin specific peptidase 1 | -2.24 | -1.44 | -1.84 |
| 55589 | BMP2K | BMP2 inducible kinase | -2.24 | -1.45 | -1.84 |
| 201895 | C4orf34 | chromosome 4 open reading frame 34 | -2.28 | -1.38 | -1.83 |
| 6713 | SQLE | squalene epoxidase | -2.19 | -1.43 | -1.81 |
| 10952 | SEC61B | Sec61 beta subunit | -1.98 | -1.61 | -1.79 |
| 7171 | TPM4 | tropomyosin 4 | -2.26 | -1.28 | -1.77 |
| 83990 | BRIP1 | BRCA1 interacting protein C-terminal helicase 1 | -1.86 | -1.68 | -1.77 |
| 220930 | LOC220930 | hypothetical LOC220930 | -1.75 | -1.78 | -1.76 |
| 10186 | LHFP | lipoma HMGIC fusion partner | -1.90 | -1.63 | -1.76 |
| 1389 | CREBL2 | cAMP responsive element binding protein-like 2 | -2.18 | -1.34 | -1.76 |
| 857 | CAV1 | caveolin 1, caveolae protein, 22kDa | -2.19 | -1.28 | -1.74 |
| 10944 | C11orf58 | chromosome 11 open reading frame 58 | -1.78 | -1.68 | -1.73 |
| 7168 | TPM1 | tropomyosin 1 (alpha) | -1.88 | -1.58 | -1.73 |
| 64065 | PERP | PERP, TP53 apoptosis effector | -2.20 | -1.24 | -1.72 |
| 4478 | MSN | moesin | -2.01 | -1.37 | -1.69 |
| 79026 | AHNAK | AHNAK nucleoprotein | -2.15 | -1.23 | -1.69 |
| 6715 | SRD5A1 | steroid-5-alpha-reductase, alpha polypeptide 1 (3-oxo-5 alpha-steroid delta 4-dehydrogenase alpha 1) | -1.73 | -1.63 | -1.68 |
| 56005 | C19orf10 | chromosome 19 open reading frame 10 | -1.97 | -1.38 | -1.68 |
| 54842 | MFSD6 | major facilitator superfamily domain containing 6 | -2.12 | -1.23 | -1.67 |
| 2512 | FTL | ferritin, light polypeptide | -2.01 | -1.27 | -1.64 |
| 55425 | KIAA1704 | KIAA1704 | -1.85 | -1.39 | -1.62 |
| 65977 | PLEKHA3 | pleckstrin homology domain containing, family A (phosphoinositide binding specific) member 3 | -2.01 | -1.22 | -1.61 |
| 79001 | VKORC1 | vitamin K epoxide reductase complex, subunit 1 | -1.89 | -1.28 | -1.59 |
| 285362 | SUMF1 | sulfatase modifying factor 1 | -1.81 | -1.36 | -1.58 |
| 665 | BNIP3L | BCL2/adenovirus E1B 19kDa interacting protein 3-like | -1.45 | -1.68 | -1.56 |
| 51661 | FKBP7 | FK506 binding protein 7 | -1.39 | -1.72 | -1.56 |
| 4833 | NME4 | non-metastatic cells 4, protein expressed in | -1.95 | -1.13 | -1.54 |
| 203068 | TUBB | tubulin, beta | -1.72 | -1.35 | -1.54 |
| 1362 | CPD | carboxypeptidase D | -1.63 | -1.43 | -1.53 |
| 201725 | C4orf46 | chromosome 4 open reading frame 46 | -1.47 | -1.58 | -1.53 |
| 493869 | GPX8 | glutathione peroxidase 8 (putative) | -1.47 | -1.57 | -1.52 |
| 51304 | ZDHHC3 | zinc finger, DHHC-type containing 3 | -1.68 | -1.36 | -1.52 |
| 100128203 | ENSAP3 | endosulfine alpha pseudogene 3 | -1.96 | -1.07 | -1.51 |
| 125950 | RAVER1 | ribonucleoprotein, PTB-binding 1 | -1.67 | -1.36 | -1.51 |
| 91750 | LIN52 | lin-52 homolog (C. elegans) | -1.90 | -1.10 | -1.50 |
| 124152 | IQCK | IQ motif containing K | -1.55 | -1.45 | -1.50 |
| 9208 | LRRFIP1 | leucine rich repeat (in FLII) interacting protein 1 | -1.77 | -1.23 | -1.50 |
| 3920 | LAMP2 | lysosomal-associated membrane protein 2 | -1.38 | -1.62 | -1.50 |
| 54839 | LRRC49 | leucine rich repeat containing 49 | -1.32 | -1.67 | -1.49 |
| 286133 | SCARA5 | scavenger receptor class A, member 5 (putative) | -1.49 | -1.48 | -1.48 |
| 11189 | CELF3 | CUGBP, Elav-like family member 3 | -1.74 | -1.22 | -1.48 |
| 7732 | RNF112 | ring finger protein 112 | -1.77 | -1.19 | -1.48 |
| 1783 | DYNC1LI2 | dynein, cytoplasmic 1, light intermediate chain 2 | -1.70 | -1.25 | -1.47 |
| 55893 | ZNF395 | zinc finger protein 395 | -1.67 | -1.27 | -1.47 |
| 10970 | CKAP4 | cytoskeleton-associated protein 4 | -1.88 | -1.03 | -1.46 |
| 100131727 | LOC100131727 | hypothetical LOC100131727 | -1.87 | -1.03 | -1.45 |
| 283373 | ANKRD52 | ankyrin repeat domain 52 | -1.73 | -1.15 | -1.44 |
| 1070 | CETN3 | centrin, EF-hand protein, 3 (CDC31 homolog, yeast) | -1.24 | -1.62 | -1.43 |
| 730101 | LOC730101 | hypothetical LOC730101 | -1.57 | -1.29 | -1.43 |
| 2760 | GM2A | GM2 ganglioside activator | -1.42 | -1.44 | -1.43 |
| 114971 | PTPMT1 | protein tyrosine phosphatase, mitochondrial 1 | -1.45 | -1.40 | -1.43 |
| 4860 | PNP | purine nucleoside phosphorylase | -1.31 | -1.54 | -1.42 |
| 100130856 | LOC100130856 | hypothetical LOC100130856 | -1.62 | -1.20 | -1.41 |
| 63920 | C5orf54 | chromosome 5 open reading frame 54 | -1.19 | -1.63 | -1.41 |
| 100130733 | LRRC70 | leucine rich repeat containing 70 | -1.54 | -1.28 | -1.41 |
| 55345 | C4orf21 | chromosome 4 open reading frame 21 | -1.44 | -1.38 | -1.41 |
| 1265 | CNN2 | calponin 2 | -1.41 | -1.38 | -1.40 |
| 375346 | TMEM110 | transmembrane protein 110 | -1.74 | -1.05 | -1.39 |
| 29979 | UBQLN1 | ubiquilin 1 | -1.35 | -1.43 | -1.39 |
| 8450 | CUL4B | cullin 4B | -1.50 | -1.27 | -1.39 |
| 252983 | STXBP4 | syntaxin binding protein 4 | -1.40 | -1.35 | -1.37 |
| 204 | AK2 | adenylate kinase 2 | -1.36 | -1.39 | -1.37 |
| 55300 | PI4K2B | phosphatidylinositol 4-kinase type 2 beta | -1.39 | -1.31 | -1.35 |
| 26140 | TTLL3 | tubulin tyrosine ligase-like family, member 3 | -1.46 | -1.24 | -1.35 |
| 286151 | FBXO43 | F-box protein 43 | -1.38 | -1.29 | -1.33 |
| 10632 | ATP5L | ATP synthase, H+ transporting, mitochondrial F0 complex, subunit G | -1.47 | -1.18 | -1.33 |
| 221336 | BEND6 | BEN domain containing 6 | -1.46 | -1.18 | -1.32 |
| 5819 | PVRL2 | poliovirus receptor-related 2 (herpesvirus entry mediator B) | -1.57 | -1.06 | -1.32 |
| 267020 | ATP5L2 | ATP synthase, H+ transporting, mitochondrial F0 complex, subunit G2 | -1.49 | -1.14 | -1.31 |
| 10746 | MAP3K2 | mitogen-activated protein kinase kinase kinase 2 | -1.37 | -1.26 | -1.31 |
| 10447 | FAM3C | family with sequence similarity 3, member C | -1.19 | -1.44 | -1.31 |
| 80727 | TTYH3 | tweety homolog 3 (Drosophila) | -1.55 | -1.08 | -1.31 |
| 23468 | CBX5 | chromobox homolog 5 (HP1 alpha homolog, Drosophila) | -1.28 | -1.34 | -1.31 |
| 7153 | TOP2A | topoisomerase (DNA) II alpha 170kDa | -1.60 | -1.01 | -1.31 |
| 10615 | SPAG5 | sperm associated antigen 5 | -1.58 | -1.03 | -1.30 |
| 9985 | REC8 | REC8 homolog (yeast) | -1.44 | -1.17 | -1.30 |
| 8720 | MBTPS1 | membrane-bound transcription factor peptidase, site 1 | -1.28 | -1.32 | -1.30 |
| 200232 | C20orf106 | chromosome 20 open reading frame 106 | -1.30 | -1.29 | -1.29 |
| 29766 | TMOD3 | tropomodulin 3 (ubiquitous) | -1.57 | -1.01 | -1.29 |
| 5352 | PLOD2 | procollagen-lysine, 2-oxoglutarate 5-dioxygenase 2 | -1.48 | -1.08 | -1.28 |
| 4284 | MIP | major intrinsic protein of lens fiber | -1.21 | -1.34 | -1.28 |
| 4323 | MMP14 | matrix metallopeptidase 14 (membrane-inserted) | -1.01 | -1.54 | -1.28 |
| 9656 | MDC1 | mediator of DNA-damage checkpoint 1 | -1.49 | -1.06 | -1.27 |
| 80179 | MYO19 | myosin XIX | -1.34 | -1.21 | -1.27 |
| 80726 | KIAA1683 | KIAA1683 | -1.01 | -1.53 | -1.27 |
| 4627 | MYH9 | myosin, heavy chain 9, non-muscle | -1.52 | -1.01 | -1.26 |
| 286144 | C8orf83 | chromosome 8 open reading frame 83 | -1.19 | -1.33 | -1.26 |
| 8698 | S1PR4 | sphingosine-1-phosphate receptor 4 | -1.44 | -1.05 | -1.25 |
| 440918 | FLJ46875 | hypothetical LOC440918 | -1.46 | -1.03 | -1.25 |
| 439921 | MXRA7 | matrix-remodelling associated 7 | -1.36 | -1.13 | -1.24 |
| 5780 | PTPN9 | protein tyrosine phosphatase, non-receptor type 9 | -1.38 | -1.10 | -1.24 |
| 594 | BCKDHB | branched chain keto acid dehydrogenase E1, beta polypeptide | -1.26 | -1.21 | -1.24 |
| 359821 | MRPL42P5 | mitochondrial ribosomal protein L42 pseudogene 5 | -1.41 | -1.03 | -1.22 |
| 60592 | SCOC | short coiled-coil protein | -1.30 | -1.13 | -1.21 |
| 2744 | GLS | glutaminase | -1.32 | -1.11 | -1.21 |
| 81558 | FAM117A | family with sequence similarity 117, member A | -1.13 | -1.30 | -1.21 |
| 89122 | TRIM4 | tripartite motif-containing 4 | -1.10 | -1.32 | -1.21 |
| 22908 | SACM1L | SAC1 suppressor of actin mutations 1-like (yeast) | -1.17 | -1.24 | -1.21 |
| 10397 | NDRG1 | N-myc downstream regulated 1 | -1.36 | -1.04 | -1.20 |
| 7424 | VEGFC | vascular endothelial growth factor C | -1.16 | -1.22 | -1.19 |
| 171546 | C14orf147 | chromosome 14 open reading frame 147 | -1.36 | -1.02 | -1.19 |
| 6659 | SOX4 | SRY (sex determining region Y)-box 4 | -1.23 | -1.15 | -1.19 |
| 440585 | FAM183A | family with sequence similarity 183, member A | -1.04 | -1.31 | -1.17 |
| 160140 | C11orf65 | chromosome 11 open reading frame 65 | -1.04 | -1.28 | -1.16 |
| 79882 | ZC3H14 | zinc finger CCCH-type containing 14 | -1.19 | -1.13 | -1.16 |
| 631 | BFSP1 | beaded filament structural protein 1, filensin | -1.17 | -1.14 | -1.15 |
| 10552 | ARPC1A | actin related protein 2/3 complex, subunit 1A, 41kDa | -1.23 | -1.07 | -1.15 |
| 160335 | TMTC2 | transmembrane and tetratricopeptide repeat containing 2 | -1.17 | -1.13 | -1.15 |
| 5983 | RFC3 | replication factor C (activator 1) 3, 38kDa | -1.02 | -1.26 | -1.14 |
| 124454 | EARS2 | glutamyl-tRNA synthetase 2, mitochondrial (putative) | -1.04 | -1.23 | -1.14 |
| 54585 | LZTFL1 | leucine zipper transcription factor-like 1 | -1.17 | -1.09 | -1.13 |
| 582 | BBS1 | Bardet-Biedl syndrome 1 | -1.04 | -1.22 | -1.13 |
| 84153 | RNASEH2C | ribonuclease H2, subunit C | -1.10 | -1.15 | -1.13 |
| 146779 | EFCAB3 | EF-hand calcium binding domain 3 | -1.08 | -1.17 | -1.12 |
| 10949 | HNRNPA0 | heterogeneous nuclear ribonucleoprotein A0 | -1.20 | -1.05 | -1.12 |
| 56267 | CCBL2 | cysteine conjugate-beta lyase 2 | -1.22 | -1.02 | -1.12 |
| 594 | BCKDHB | branched chain keto acid dehydrogenase E1, beta polypeptide | -1.18 | -1.06 | -1.12 |
| 100292409 | LOC100292409 | hypothetical protein LOC100292409 | -1.11 | -1.12 | -1.11 |
| 10054 | UBA2 | ubiquitin-like modifier activating enzyme 2 | -1.09 | -1.14 | -1.11 |
| 6228 | RPS23 | ribosomal protein S23 | -1.09 | -1.13 | -1.11 |
| 10229 | COQ7 | coenzyme Q7 homolog, ubiquinone (yeast) | -1.19 | -1.01 | -1.10 |
| 55142 | HAUS2 | HAUS augmin-like complex, subunit 2 | -1.19 | -1.00 | -1.09 |
| 314 | AOC2 | amine oxidase, copper containing 2 (retina-specific) | -1.02 | -1.17 | -1.09 |
| 1211 | CLTA | clathrin, light chain (Lca) | -1.12 | -1.03 | -1.08 |
| 23362 | PSD3 | pleckstrin and Sec7 domain containing 3 | -1.13 | -1.02 | -1.07 |
| 221016 | CCDC7 | coiled-coil domain containing 7 | -1.07 | -1.07 | -1.07 |
| 54414 | SIAE | sialic acid acetylesterase | -1.06 | -1.07 | -1.06 |
| 53340 | SPA17 | sperm autoantigenic protein 17 | -1.00 | -1.11 | -1.06 |
| 730631 | LOC730631 | hypothetical LOC730631 | -1.03 | -1.06 | -1.05 |
| 10776 | ARPP19 | cAMP-regulated phosphoprotein, 19kDa | -1.07 | -1.02 | -1.04 |
| 153577 | LOC153577 | hypothetical protein LOC153577 | -1.04 | -1.04 | -1.04 |
| 91869 | RFT1 | RFT1 homolog (S. cerevisiae) | -1.03 | -1.04 | -1.03 |
| 23596 | OPN3 | opsin 3 | -1.04 | -1.02 | -1.03 |
