# Supplementary material for: Tumour suppressors miR-1 and miR-133a target the oncogenic function of purine nucleoside phosphorylase (PNP) in prostate cancer
Source: Br J Cancer. 2011 Nov 8;106(2):405–13. doi: 10.1038/bjc.2011.462 (PMC3261671; doi:10.1038/bjc.2011.462)
Supplement: Supplementary Table 3 [file bjc2011462x6.ppt]

## Slide 1
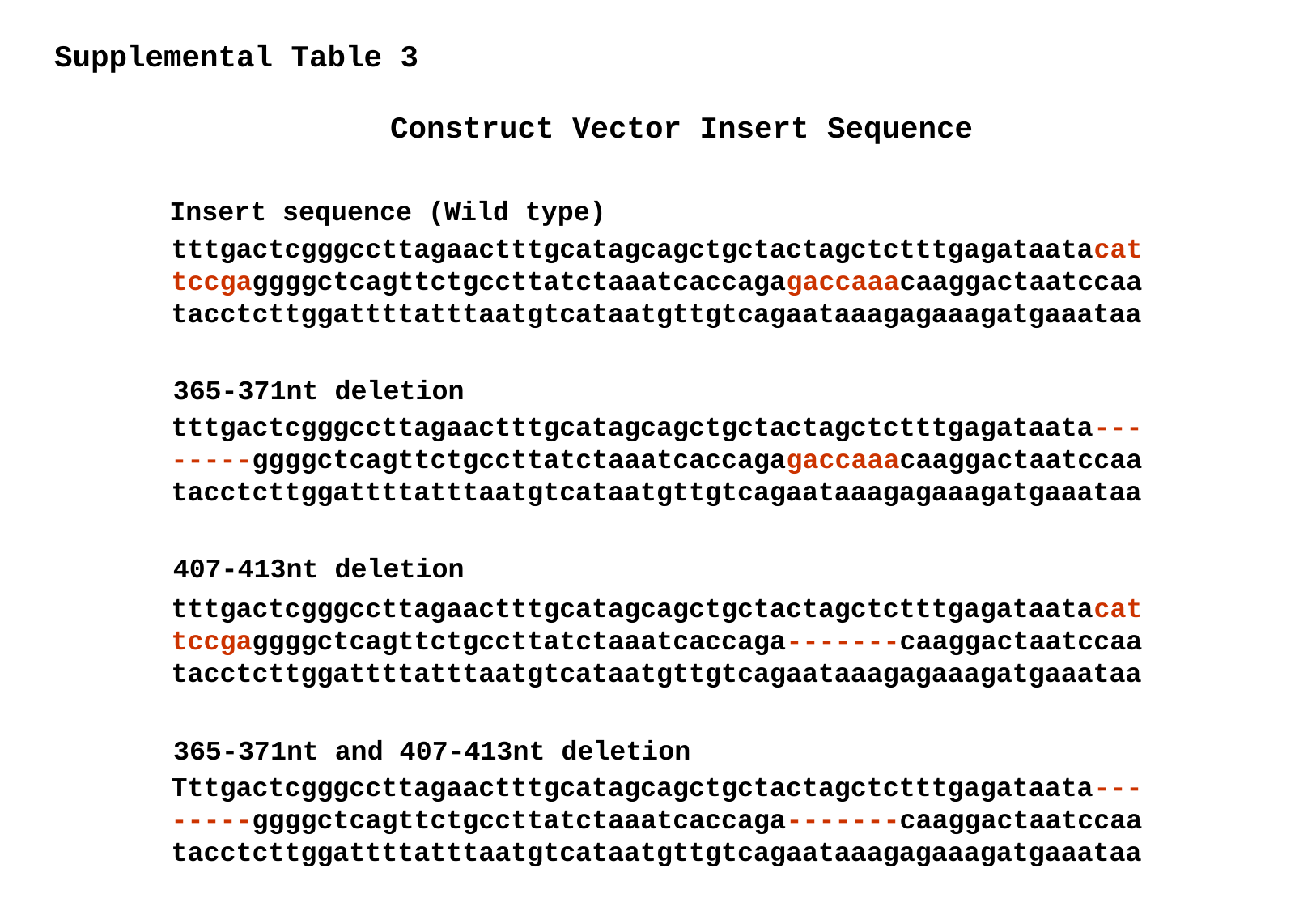

Supplemental Table 3
 Construct Vector Insert Sequence
Insert sequence (Wild type)
tttgactcgggccttagaactttgcatagcagctgctactagctctttgagataatacat
tccgaggggctcagttctgccttatctaaatcaccagagaccaaacaaggactaatccaa
tacctcttggattttatttaatgtcataatgttgtcagaataaagagaaagatgaaataa
365-371nt deletion
tttgactcgggccttagaactttgcatagcagctgctactagctctttgagataata---
-----ggggctcagttctgccttatctaaatcaccagagaccaaacaaggactaatccaa
tacctcttggattttatttaatgtcataatgttgtcagaataaagagaaagatgaaataa
407-413nt deletion
tttgactcgggccttagaactttgcatagcagctgctactagctctttgagataatacat
tccgaggggctcagttctgccttatctaaatcaccaga-------caaggactaatccaa
tacctcttggattttatttaatgtcataatgttgtcagaataaagagaaagatgaaataa
365-371nt and 407-413nt deletion
Tttgactcgggccttagaactttgcatagcagctgctactagctctttgagataata---
-----ggggctcagttctgccttatctaaatcaccaga-------caaggactaatccaa
tacctcttggattttatttaatgtcataatgttgtcagaataaagagaaagatgaaataa
